# N6-methyladenosine-modified circRIMS2 mediates synaptic and memory impairments by activating GluN2B ubiquitination in Alzheimer's disease

**DOI:** 10.1186/s40035-023-00386-6

**Published:** 2023-11-28

**Authors:** Xiong Wang, Jiazhao Xie, Lu Tan, Yanjun Lu, Na Shen, Jiaoyuan Li, Hui Hu, Huijun Li, Xiaoguang Li, Liming Cheng

**Affiliations:** 1 Department of Laboratory Medicine, Tongji Hospital, Tongji Medical College, Huazhong University of Science and Technology, Wuhan, 430030 China; 2 Departments of Pathophysiology, Guangxi Medical University, Nanning, 530021 China; 3 Department of Pathophysiology, School of Basic Medicine, Key Laboratory of Education Ministry of China/Hubei Province for Neurological Disorders, Tongji Medical College, Huazhong University of Science and Technology, Wuhan, 430030 China; 4 Hepatic Biliary Pancreatic Surgery Department, The Central Hospital of Wuhan, Tongji Medical College, Huazhong University of Science and Technology, Wuhan, 430014 China; 5 Clinic Center of Human Gene Research, Union Hospital, Tongji Medical College, Huazhong University of Science and Technology, Wuhan, 430022 China

**Keywords:** Alzheimer’s disease, circRNA, Synaptic dysfunction, GluN2B, Ubiquitination

## Abstract

**Background:**

Synaptic degeneration occurs in the early stage of Alzheimer's disease (AD) before devastating symptoms, strongly correlated with cognitive decline. Circular RNAs (circRNAs) are abundantly enriched in neural tissues, and aberrant expression of circRNAs precedes AD symptoms, significantly correlated with clinical dementia severity. However, the direct relationship between circRNA dysregulation and synaptic impairment in the early stage of AD remains poorly understood.

**Methods:**

Hippocampal whole-transcriptome sequencing was performed to identify dysregulated circRNAs and miRNAs in 4-month-old wild-type and APP/PS1 mice. RNA antisense purification and mass spectrometry were utilized to unveil interactions between circRIMS2 and methyltransferase 3, N6-adenosine-methyltransferase complex catalytic subunit (METTL3). The roles of circRIMS2/miR-3968 in synaptic targeting of UBE2K-mediated ubiquitination of GluN2B subunit of NMDA receptor were evaluated via numerous lentiviruses followed by morphological staining, co-immunoprecipitation and behavioral testing. Further, a membrane-permeable peptide was used to block the ubiquitination of K1082 on GluN2B in AD mice.

**Results:**

circRIMS2 was significantly upregulated in 4-month-old APP/PS1 mice, which was mediated by METTL3-dependent N6-methyladenosine (m6A) modification. Overexpression of circRIMS2 led to synaptic and memory impairments in 4-month-old C57BL/6 mice. MiR-3968/UBE2K was validated as the downstream of circRIMS2. Elevated UBE2K induced synaptic dysfunction of AD through ubiquitinating K1082 on GluN2B. Silencing METTL3 or blocking the ubiquitination of K1082 on GluN2B with a short membrane-permeable peptide remarkably rescued synaptic dysfunction in AD mice.

**Conclusions:**

In conclusion, our study demonstrated that m6A-modified circRIMS2 mediates the synaptic and memory impairments in AD by activating the UBE2K-dependent ubiquitination and degradation of GluN2B via sponging miR-3968, providing novel therapeutic strategies for AD.

**Supplementary Information:**

The online version contains supplementary material available at 10.1186/s40035-023-00386-6.

## Introduction

Alzheimer's disease (AD), the leading cause of dementia, is responsible for over 60% of all dementia cases. AD is also the sixth leading cause of death. The deaths related to AD increased by more than 145% between 2000 and 2019 in the United States, and this increase was further exacerbated by the COVID-19 pandemic [1]. The prominent features of AD include synaptic degeneration, tau tangles, and amyloid-β (Aβ) deposition. Synaptic degeneration occurs in the early stage of AD before other devastating symptoms occur, and is strongly correlated with cognitive decline [2]. Both tau and Aβ contribute to synaptic degeneration in AD, and most anti-Aβ and anti-tau therapies have so far failed [3]. Strategies targeting synapses to ameliorate synaptic structural change, damage, and synaptic loss, have shown promise in rescuing cognitive impairments [4]. Thus, uncovering the mechanisms of synaptic dysfunction in AD may lead to development of novel therapeutic strategies.

Circular RNAs (circRNAs) are naturally occurring RNA circles formed by back-splicing of host genes that generate protein-coding mRNAs via canonical splicing. They are abundantly enriched in neural tissues, specifically derived from synapse-related genes, and their expression is regulated by synaptic plasticity [5, 6]. The functional mechanisms of circRNAs include sponging miRNAs or RNA-binding proteins, and serving as a translational template to produce peptides [7]. Aberrant expression of circRNAs occurs in the early stage of AD, and is significantly correlated with AD diagnosis, neuropathological severity, and clinical dementia severity [5]. The circRNA expression pattern and the circRNA/miRNA competing endogenous RNA (ceRNA) network have been extensively investigated in both AD patients and animal models across different brain regions and stages [5, 8–10]. Certain circRNAs play essential roles in regulating Aβ deposition, tau phosphorylation, and neuroinflammation [11–14]. For instance, Song et al. [14] reported that circCwc27 interacts with purine-rich element-binding protein A (Pur-α), halts nuclear transport of Pur-α, reduces Pur-α binding to the promoter of amyloid precursor protein (APP), and leads to Aβ deposition and memory impairment in 6-month-old APP/PS1 mice. Moreover, blood circRNAs have been reported as promising biomarkers for AD diagnosis [15, 16]. However, the direct link between circRNA dysregulation and synaptic impairment in the early stage of AD is not clear.

In this study, we screened circRNA changes in the hippocampus of 4-month-old APP/PS1 (APPswe and PSEN1dE9) mice, an early stage with synaptic dysfunction and without obvious Aβ deposition [17]. We hope to clarify the associations between circRIMS2 and AD learning and memory impairment, and illustrate the molecular mechanisms through behavioral testing, fluorescence imaging and mass spectrometry technologies.

## Methods

### Animals

Male APP/PS1 (APPswe and PSEN1dE9) mice were purchased from the Animal Model Research Center of Nanjing University (Nanjing, China) and maintained by Gene&PeacebiotechCo., Ltd., (Guangzhou, China). All procedures were approved by the Animal Care and Use Committee of Tongji Hospital, Tongji Medical College of Huazhong University of Science and Technology.

### Plasmids

The whole circRIMS2 sequence was cloned into the pcircRNA-1 circRNA overexpression vector (Additional file 1: Fig. S1a) purchased from BersinBio (Guangzhou, China).

### RNA sequencing

Total RNA was isolated from hippocampus with TRIzol reagent (Thermo Fisher Scientific, Carlsbad, CA), and was quantified with Qubit 3.0 Spectrophotometer (Thermo Fisher Scientific). The sequencing libraries for mRNA and circRNA were generated by TruSeq Stranded Total RNA Library Prep Kit (Illumina, San Diego, CA). The sequencing libraries for miRNAs were generated by TruSeq small RNA sample Preparation kit (Illumina). The libraries were sequenced on a Hiseq 2500 platform (Illumina). Clean reads were aligned to the reference genome mm10 using HISAT2.

Unmapped reads were used to identify circRNAs using CIRI2. miRBase was used as reference to find known or novel miRNAs with miRDeep2. The mapped reads of mRNA were assembled by Stringtie.

### Aβ oligomer preparation

Aβ42 oligomers were prepared as described previously [18]. Aβ42 peptide (Chinapeptides, Wuhan, China) was dissolved in 100% hexafluoroisopropanol at 1 mM, followed by vacuum to remove hexafluoroisopropanol. The peptide was resuspended in dimethyl sulfoxide at a concentration of 5 mM, diluted to 100 μM with F12 culture medium, and further incubated for 24 h at 4 °C. The solution was centrifuged for 20 min at 13,000 rpm, and the supernatant was used.

### RNA antisense purification (RAP) assay

For RAP assay, an RAP Kit (BersinBio, Guangzhou, China) was used following the manufacturer's protocol. Biotin-labeled circRIMS2 probe (biotin-5′-TGATCCCTGGACACTGATGGACTTCGTTTTTTCTCTCCAATGTTCCCTTTGGTGGAAGCT-3′-biotin) and scramble control probe (biotin-5′-GCCTGATGCGGTATTTTCTCCTTACGCATCTGTGCGGTATTTCACACCGCATATGGTGCA-3′-biotin) were synthesized by BersinBio (Guangzhou, China). Briefly, cross-linked cells were lysed, sonicated, and then hybridized with the probe for 4 h at 37 °C. The hybridization mixture was then treated with C1 magnetic beads (Invitrogen, Carlsbad, CA) for 1 h. After that, the bound proteins were cleaned and prepared for silver staining and mass spectrometry analysis.

### Morris water maze (MWM)

A platform was placed below the water surface in one of the quadrants of the maze. Each mouse underwent five consecutive days of training, with three trials each day. At the beginning of each trial, a mouse was placed in one quadrant, and the trial ended once it found the platform and stayed there for at least 3 s. If the mouse failed to find the platform in one minute, it was manually led to the platform and allowed to stay on it for 15 s. On day 7, after a day of rest, the mice were tested. A video camera (Techman, Chengdu, China) was placed 2 m above the water surface to capture the swimming path, the latency to find the platform, the duration in the target zone, and the speed.

### Novel object recognition (NOR) test

One day before the habituation test, the mice were placed in the arena without any objects for 5 min. On the training day, two objects, designated A and B, were introduced to the arena and the mice were allowed 5 min to explore. After the training session, the equipment and arena were thoroughly cleaned with 75% (*v*/*v*) ethanol. On the testing day, subject B was replaced with a brand-new subject C. A video camera was used to capture mouse behavior, and data were analyzed with the Any-maze behavior tracking software from Techman. The times the mice touched objects A and C were recorded as TA and TC, respectively. The preference for unfamiliar objects was calculated by dividing TC by the total exploration time (TA + TC).

### Golgi-cox staining

After anesthesia with 2% pentobarbital sodium, the mouse brains were removed and immersed in Golgi staining solutions A/B for 14 days in darkness. Subsequently, the solutions A/B were replaced with solution C for 7 days at 4 °C in the dark. The 100-µm brain slices were prepared using the VT1200S vibratome (Leica, Wetzlar, Germany), and scanned using the VS120 microscope (Olympus, Tokyo, Japan) for the Sholl analysis, and the spine images were scanned using the Ni-E microscope (Nikon, Tokyo, Japan). The spine counts and Sholl analysis were assessed using ImageJ software (NIH, Bethesda, MD).

### Western blotting (WB) and co-immunoprecipitation (Co-IP)

Cultured N2a cells were pre-treated with 10 µM of MG132 for 30 min to examine the ubiquitination of GluN2B following immunoprecipitation. The cells were further lysed with RIPA lysis buffer for 10 min, and supernatant was collected and treated with A + G agarose for 30 min at 4 °C, followed by centrifugation at 8000× *g* for 10 min at 4 °C. The supernatant was then incubated at 4 °C for 12–16 h with specified primary antibodies (GluN2B) and protein A + G agarose for co-immunoprecipitation. For tissue or cell Co-IP, supernatants were collected, treated with A + G agarose at 4 °C for 30 min, then centrifuged at 8000× *g* for 10 min at 4 °C. Then, the supernatants were incubated at 4 °C for 12–16 h with the primary antibody for UBE2K or GluN2B and protein A + G agarose. The agarose beads were washed, resuspended in 40 µl of SDS buffer, and further heated at 95 °C for 10 min. The supernatants were examined by WB [19].

Total protein concentrations were measured by the bicinchoninic acid assay. Protein samples were loaded onto 8%–15% SDS-PAGE gels, and transferred to PVDF membranes (Merck Millipore, Danvers, MA). The membranes were blocked with 5% non-fat milk before overnight incubation with primary antibodies. On the next day, the membranes were incubated with HRP-conjugated secondary antibodies at 25 °C. The blots were imaged using ECL substrate in the ChemiScope 6000 luminometer (Clinx, Shanghai, China) and analyzed using the ImageJ software. The antibodies are listed in Additional file 1: Table S1.

### RNA isolation and real-time quantitative PCR (qRT-PCR)

RNA was isolated from cells or the hippocampus using TRIzol reagent. cDNA was synthesized with the cDNA Synthesis Kit (Yeasen, Shanghai, China), and qPCR experiments were conducted using the SYBR mix (TAKARA, Japan) in the ABI7500 machine (Applied Biosystem, Pleasanton, CA). The qPCR system included 1 μl of cDNA, 5 μl of SYBR Green master mix, and 0.5 μM of forward and reverse primers. The expression of circRNAs and miRNAs was normalized to GAPDH and U6, respectively. The primers used for qPCR were as follows: miR-3968-RT: GTCGTATCCAGTGCAGGGTCCGAGGTATTCGCACTGGATACGACTGGTGT, miR-3968-F: CGAATCCCACTCCAGACACCA, miR-3968-R: CCAGTGCAGGGTCCGAGGTATTC; U6-RT: AAAAATATGGAACGCTTCACGAATTTG, U6-F: GTGCTCGCTTCGGCAGCACATA, U6-R: GCGCAGGGGCCATGCTAATCTTC; circRIMS2-F: ATCAAGTACTCCGGGAACAG, circRIMS2-R: CTTTCTTCACTTTGCTCGTATC; UBE2K-F: AATCAAGCGGGAGTTCAAGG, UBE2K-R: TGTCTGGAGGTCCTGCTATTTC; RIMS2-F: AGGAATACCAGGCACGCTAC, RIMS2-R: ACATCACTGTGCCTTCTCTCAT; METTL3-F: AACATCTGTGGCCCCTGAAC, METTL3-R: TGGCGTAGAGATGGCAAGAC; GAPDH-F: GGCATCTTGGGCTACACTG, GAPDH-R: GTGGAAGAGTGGGAGTTGC.

### Immunofluorescence and fluorescence in situ hybridization (FISH)

Primary mouse cortical neurons were initially seeded on glass coverslips and fixed with 4% paraformaldehyde in PBS for 30 min at 25 °C. Following this, cells were blocked with 3% BSA for 30 min at 25 °C and incubated with primary antibodies. The coverslips were washed and incubated with a secondary antibody for 1 h at 25 °C. The DAPI fluorescent dye was used to stain the nuclei. For FISH, the probes for circRIMS2 (red) and miR-3968 (green) were from Bersinbio (Guangzhou, China), and were hybridized with N2a cells at 42 °C for 20 h. The fluorescence of the cells was examined using LSM800 fluorescence microscope (Carl Zeiss, Germany).

### Stereotaxic brain injection of virus

Lentiviruses that coded for UBE2K, shUBE2K, circRIMS2, or miR3968 were purchased from Viraltherapy (Wuhan, China) and had a virus titer of 5 × 10^8^ TU/ml. Adeno-associated virus (AAV) serotype 9 that coded for shMETTL3 (CCTCCAAGATGATGCACATTT) was purchased from General Biol (Anhui) Co. Ltd. (Chuzhou, China) with a virus titer of 1E+12 vg/ml. All genes within the virus vector were promoted by the CMV-promoter. For hippocampal injection, mice were anesthetized and placed in a stereotaxic equipment, and were bilaterally injected with 1 μl of lentivirus or AAV (AP ± 2.5, DV -2.0, ML -2.0) at a rate of 1 μl/10 min. The needle of the syringe was kept in place for 10 min after injection.

### Methylated RNA immunoprecipitation-quantitative PCR (MeRIP-PCR)

RNA was isolated from the hippocampus or cells using TRIzol reagent, and 1/10 of the RNA was kept as input. Prewashed Protein A/G Magnetic Beads (Tiangen, Beijing, China) were incubated with 5 μg of either rabbit immunoglobulin G (IgG) or anti-m6A antibody (Zenbio, Chengdu, China) at 4 °C for 2 h. After three washes, pure poly(A) RNA, 1 × IP buffer, and RNase inhibitors were combined with the antibody-conjugated beads. Following proteinase K digestion, the methylated RNA was precipitated using glycogen and 3 M sodium acetate overnight. The positions of circRIMS2 sequence-based m6A modification sites were predicted using SRAMP (http://www.cuilab.cn/sramp). The m6A enrichment of circRIMS2 was calculated via normalizing to the input. Further enrichment was determined by qPCR.

### RNA immunoprecipitation

RNA immunoprecipitation was performed using an Imprint® RIP kit from Millipore. Briefly, 1 × 10^7^ N2a cells were lysed with 150 μl of RNA immunoprecipitation lysis buffer, and the cell lysates were treated with magnetic beads coated with 5 μg of specific antibodies against mouse IgG or Ago (Abcam, Boston, MA) at room temperature for 4 h. The immunoprecipitated RNA was isolated by incubating the RNA–protein complexes with proteinase K digestion buffer after six rounds of washing, and were further used for qRT-PCR.

## Results

### The circRIMS2/miR-3968 pathway is abnormally activated in several AD models

**Fig. 1:**
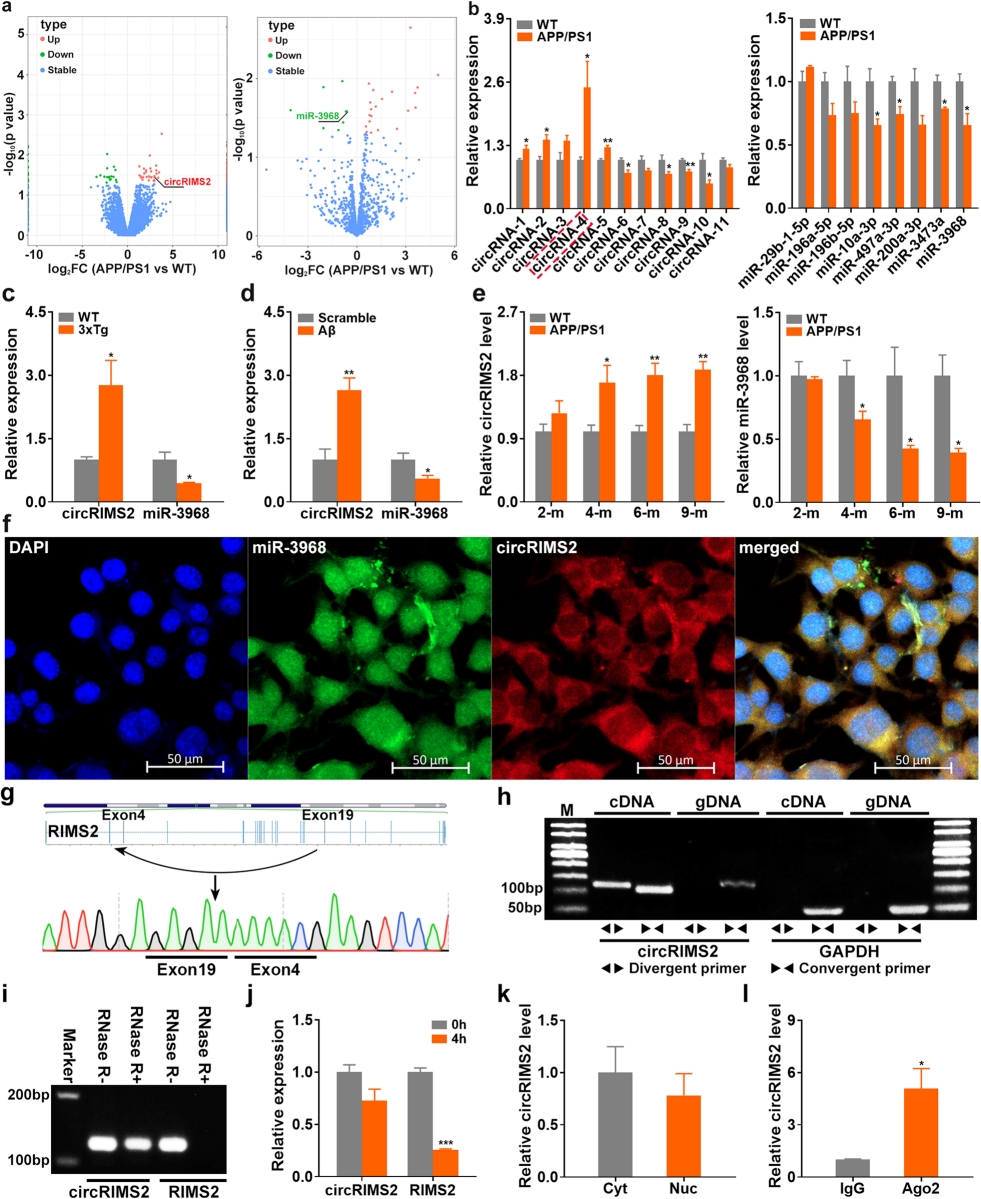
Characterization of the circRIMS2/miR-3968 pathway in AD models. **a** Volcano plots displaying 116 differentially expressed circRNAs and 29 differentially expressed miRNAs in the hippocampus of 4-month-old wild-type (WT) and APP/PS1 mice by sequencing (*n* = 3). circRIMS2 was increased while miR-3968 was decreased. The cut-off *P* value was 0.05. **b** qRT-PCR analysis of dysregulated ceRNA pairs involving 11 circRNAs (left,* n* = 4) and 8 miRNAs (right, *n* = 3) in the hippocampus of 4-month-old APP/PS1 mice. **c** qRT-PCR analysis of circRIMS2 and miR-3968 levels in the hippocampus of 4-month-old 3 × Tg and WT mice (*n* = 4). **d** qRT-PCR analysis of circRIMS2 and miR-3968 levels in scramble- and Aβ-treated mouse primary cortical neurons (*n* = 4). **e** Relative expression of circRIMS2 (left) and miR-3968 (right) in the hippocampus of APP/PS1 and WT mice at diverse stages (*n* = 4). **f** The distribution of circRIMS2 and miR-3968 in N2a cells was detected by FISH. CircRIMS2 and miR-3968 were labeled in red and green, respectively; nuclei were labeled with DAPI (blue). Scale bars, 50 μm. **g** Schematic illustration of circRIMS2 formation through the circularization of exon 4 and 19 in RIMS2. The back-splicing junction site of circRIMS2 was validated by Sanger sequencing. **h** PCR analysis of circRIMS2 and GAPDH in cDNA and genomic DNA (gDNA) amplified by convergent and divergent primers. **i** PCR analysis of circRIMS2 and RIMS2 from 3 μg RNA treated with 10U RNase R (GENESEED, Guangzhou, China) for 10 min. **j** qRT-PCR detected the abundance of circRIMS2 and RIMS2 in N2a cells treated with ActD (2 μg/ml) for 4 h (*n* = 3). **k** qRT-PCR analysis of circRIMS2 in the nucleus (Nuc) and cytoplasm (Cyt) of N2a cells (*n* = 3). Anti-Ago2 RNA immunoprecipitation was performed in N2a cells, and circRIMS2 was enriched by Ago2 (*n* = 4). Data are presented as mean ± S.E.M., and two-tailed *t*-tests were used unless otherwise specified. *P < 0.05, **P < 0.01, ***P < 0.001

Dysregulation of circRNAs occurs early in AD and precedes dementia symptoms [5]. We conducted RNA sequencing to identify dysregulated miRNAs and circRNAs in the hippocampus of 4-month-old wild-type (WT) and APP/PS1 mice. A total of 116 dysregulated circRNAs (downregulation: 43, upregulation: 73) and 29 dysregulated miRNAs (downregulation: 8, upregulation: 21) were detected in the APP/PS1 mice (Fig. 1a; Additional file 1: Tables S2–S3). The circRNA/miRNA ceRNA pairs were predicted through the miRanda software, and 14 circRNA/miRNA pairs consisting of 11 dysregulated circRNAs and 8 dysregulated miRNAs were predicted (Additional file 1: Tables S4). qRT-PCR validated an upregulated circRIMS2/miR-3968 ceRNA pair in APP/PS1 mice with upregulation of circRIMS2 and downregulation of miR-3968 (Fig. 1b). We observed similar changes in circRIMS2/miR-3968 in 3 × Tg mice and Aβ-treated primary cortical mouse neurons, suggesting that this pathway is abnormally activated in several AD models (Fig. 1c, d). We also examined changes in circRIMS2/miR-3968 in APP/PS1 mice at diverse stages and found that circRIMS2 gradually increased from 4 months, while miR-3968 showed reverse changes (Fig. 1e). FISH identified the colocalization of circRIMS2 and miR-3968 in the cytoplasm of N2a cells (Fig. 1f). Dual-luciferase reporter assay and qRT-PCR revealed that circRIMS2 suppressed miR-3968 expression (Additional file 1: Fig. S1b, c), indicating its miRNA sponge potential.

CircRIMS2 was derived from exons 4 to 19 of the regulating synaptic membrane exocytosis 2 (RIMS2) gene (Fig. 1g). Unlike linear RIMS2 transcribed by convergent primers, circRISM2 could not be amplified from gDNA and was resistant to RNase R digestion (Fig. 1h, i). Actinomycin D (ActD) was used to block new RNA synthesis, and circRIMS2 showed higher stability than linear RIMS2 (Fig. 1j). The intracellular distribution of circRIMS2 showed no significant difference between the nucleus and the cytoplasm of N2a cells (Fig. 1k). Ago2 is essential for miRNA mature and function [20], and we found that circRIMS2 was significantly enriched by Ago2 (Fig. 1l), indicating its miRNA sponge potential.

### Upregulation of circRIMS2 is mediated through METTL3-mediated m6A modification

**Fig. 2:**
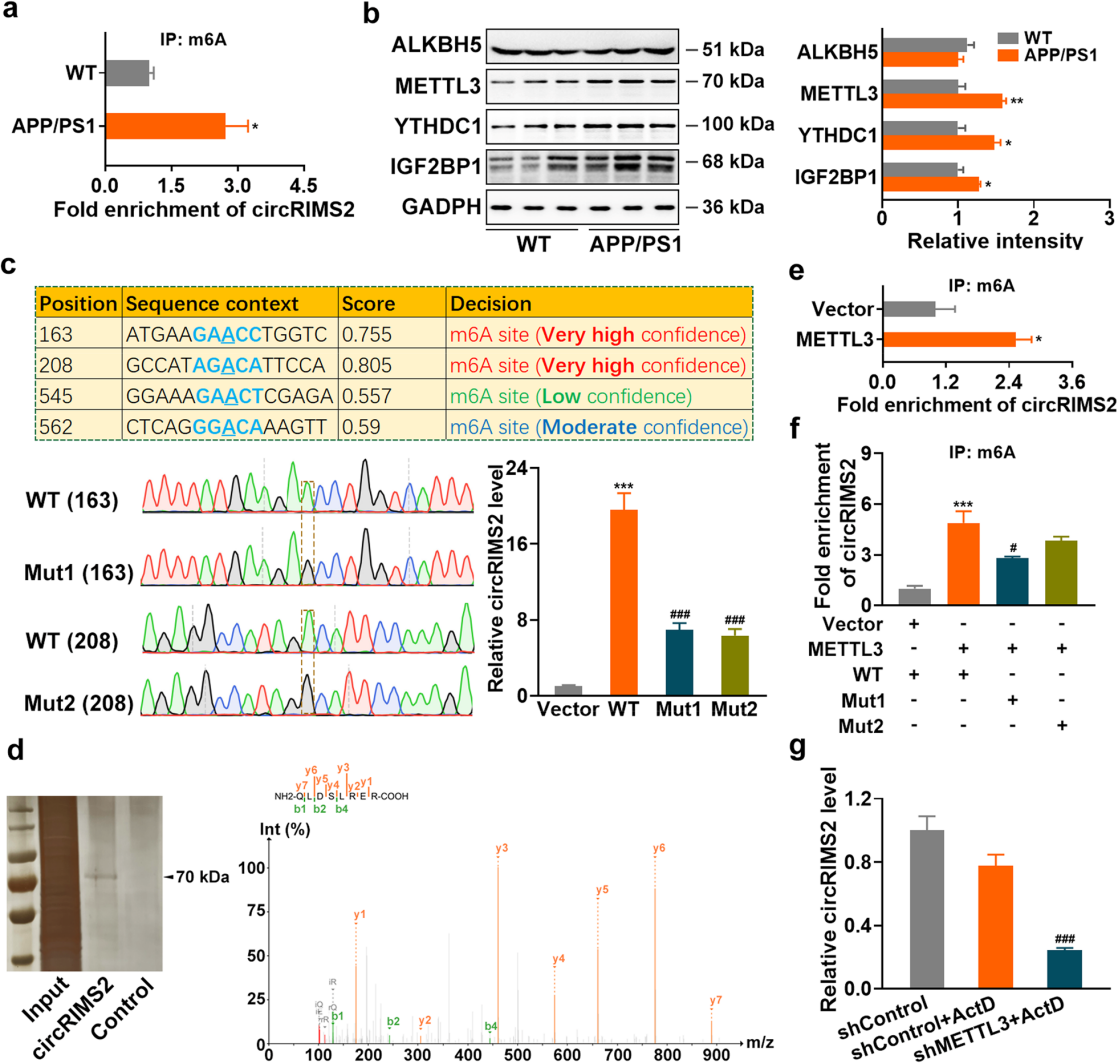
Upregulation of circRIMS2 is mediated through METTL3-dependent m6A modification. **a** The abundance of m6A-modified circRIMS2 was analyzed by MeRIP-PCR in the hippocampus of 4-month-old WT and APP/PS1 mice (*n* = 3). **b** The protein levels of ALKBH5, METTL3, YTHDC1, and IGF2BP1 were detected by WB, and quantitative analysis was performed (*n* = 3). **c** The positions of circRIMS2 sequence-based m6A modification sites were predicted using SRAMP (http://www.cuilab.cn/sramp) (upper). Sequence validation of the m6A modification sites of WT, Mut1, and Mut2 in circRIMS2 mRNA was performed by Sanger sequencing (lower left). qRT-PCR analysis of circRIMS2 was conducted after transfection with WT, Mut1, and Mut2 plasmids in N2a cells (lower right) (*n* = 5, one-way ANOVA with Tukey’s *post*-*hoc* test). **d** RNA antisense purification (RAP) was performed to screen the binding proteins of circRIMS2 (left). Specific peptide fragments of METTL3 were identified by mass spectrometry (right). **e** The abundance of m6A-modified circRIMS2 was analyzed by MeRIP-PCR in N2a cells after overexpressing METTL3 (*n* = 3). **f** The abundance of m6A-modified circRIMS2 between the vector- and METTL3-overexpressing N2a cells with WT, Mut1, or Mut2 plasmid transfection by MeRIP-PCR (*n* = 3, one-way ANOVA with Tukey’s *post-hoc* test). **g** qRT-PCR analysis of circRIMS2 was conducted in N2a cells treated with ActD for 4 h after transfection with shControl or shMETTL3 (*n* = 6, one-way ANOVA with Tukey’s *post-hoc* test). Data are presented as mean ± SEM and two-tailed t tests were used unless otherwise specified. *P < 0.05, **P < 0.01, ***P < 0.001 vs WT in** b**, vs Vector group in** c** and** e**, vs Vector+WT group in** f**. ^#^*P* < 0.05, ^###^*P* < 0.001 vs WT in** c**; vs the METTL3 + WT group in** f**; vs the shControl + ActD group in** g**

The m6A modification has been linked to the biogenesis, cytoplasmic export, function, and degradation of circRNAs [21]. The m6A level of circRIMS2 was significantly upregulated in 4-month-old APP/PS1 mice (Fig. 2a). Moreover, the m6A writer METTL3, and m6A readers YTHDC1 and IGF2BP1, were upregulated in APP/PS1 mice (Fig. 2b) and Aβ-treated primary cortical neurons (Additional file 1: Fig. S2a). Conversely, the m6A eraser ALKBH5 remained stable between APP/PS1 and WT mice. The potential m6A modification sites on circRIMS2 were predicted using SRAMP [22]. The A-to-G mutation of the two predicted m6A sites (position 163 and 208), with very high confidence, led to a remarkable decrease in circRIMS2 levels compared with the WT circRIMS2 overexpression plasmid (Fig. 2c). RNA pull-down and mass spectrometry analysis confirmed METTL3 as a putative circRIMS2-binding m6A regulator (Fig. 2d; Additional file 1: Fig. S2b). Overexpression of METTL3 increased the m6A level of circRIMS2 (Fig. 2e; Additional file 1: Fig. S2c). The A-to-G mutation of the predicted m6A sites reversed the effect of METTL3 on circRIMS2 m6A modification (Fig. 2f). Knockdown of METTL3 reversed Aβ-induced m6A modification of circRIMS2 in N2a cells and reduced the stability of circRIMS2 in ActD-treated N2a cells (Fig. 2g; Additional file 1: Fig. S2d, e). These findings suggest that METTL3-mediated m6A modification stabilizes circRIMS2.

### Overexpression of circRIMS2 induces memory and synaptic impairments in vivo

**Fig. 3:**
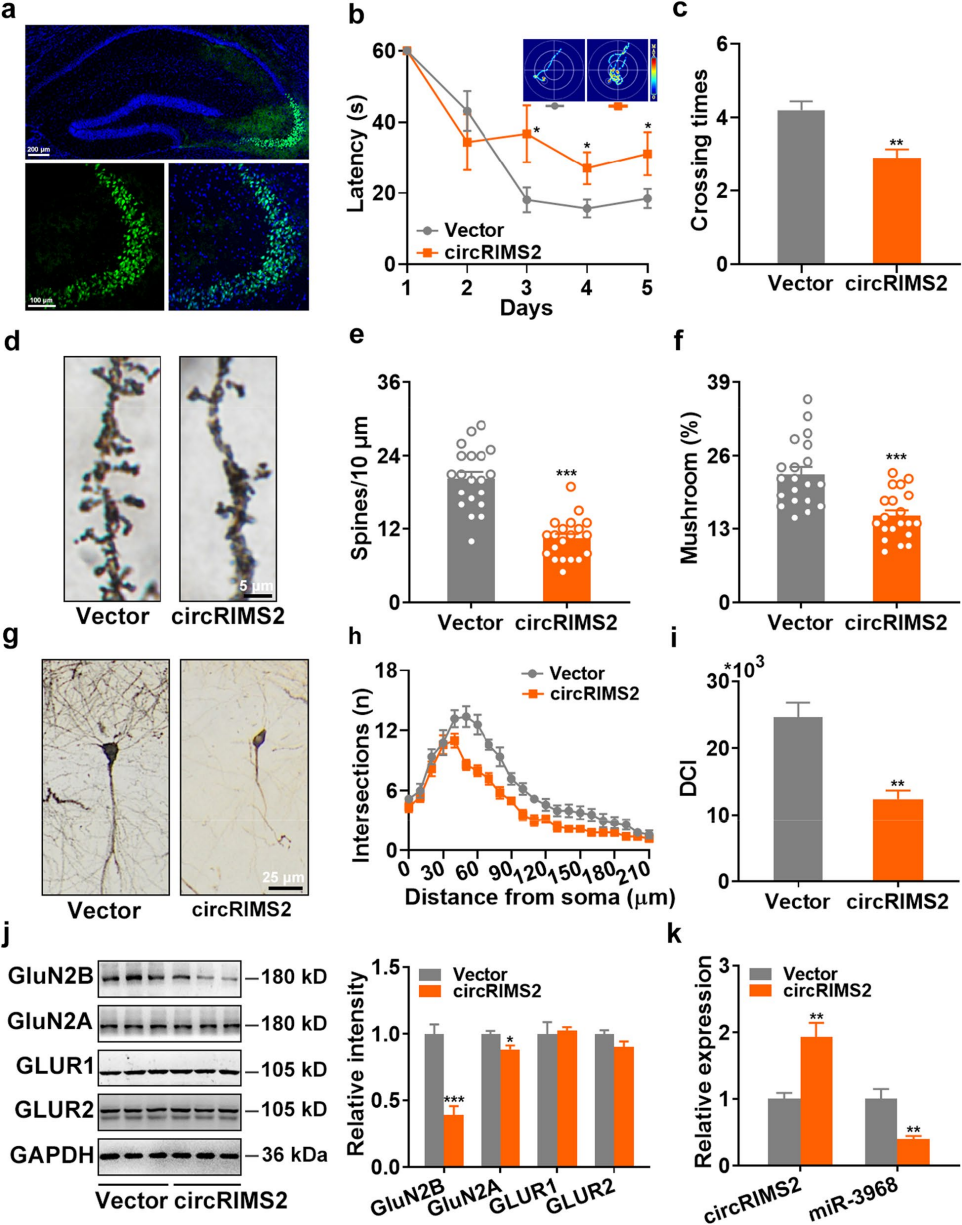
Overexpression of circRIMS2 induces memory and synaptic impairments in vivo. **a** Fluorescence images of a hippocampal slice infected with circRIMS2 lentivirus. Green represents circRIMS2 and blue represents DAPI-stained nuclei. Enlarged CA3 area is shown in the lower panel. Scale bar, 200 μm (upper) and 100 μm (lower). **b, c** MWM was conducted one month after injecting circRIMS2 or control (Vector) lentivirus into the hippocampus of 4-month-old C57BL/6 mice. Representative traces and latencies during the learning stage are shown (**b**) and crossing times (**c**) on day 7 were analyzed (*n* = 15, 10 for vector and circRIMS2, respectively). **d** Representative Golgi-cox staining images show dendritic spines of C57BL/6 mice after injection with circRIMS2 or vector lentivirus. Scale bar, 5 μm. **e, f** Changes of spine density (**e**) and percentage of mushroom spines (**f**) were assessed (*n* = 20). **g** Golgi-cox staining images show dendritic trees in circRIMS2- or vector-injected mice. Scale bar, 25 μm. **h, i** The dendritic complexity of circRIMS2- or vector-injected mice was examined using Sholl (**h**) and dendritic complexity index (DCI, **i**) (*n* = 5). **j** Protein levels of GluN2B, GluN2A, GLUR1, and GLUR2 were measured in vector- or circRIMS2-injected mice. Representative blots (left) and quantitative analysis (right) are presented (*n* = 5). **k** qRT-PCR analysis of circRIMS2 and miR-3968 levels in these mice (*n* = 4). Data are presented as mean ± SEM and two-tailed *t* tests were used unless otherwise specified. *P < 0.05, **P < 0.01, ***P < 0.001

To illustrate the involvement of circRIMS2 in the pathogenesis of AD, a lentivirus containing circRIMS2 (5 × 10^8^ TU/ml) was bilaterally injected into the hippocampus of 4-month-old C57BL/6 mice (Fig. 3a). Mice overexpressing circRIMS2 exhibited longer latency during the learning stage and fewer crossing times in the hidden platform region in the MWM during the test stage (Fig. 3b, c). Additionally, we examined synaptic plasticity in these mice by Golgi staining and observed reductions in both the dendritic spine density and the percentage of mushroom-type spines (Fig. 3d–f). Furthermore, the Sholl analysis and DCI scores indicated a decrease in dendritic complexity in mice overexpressing circRIMS2 (Fig. 3g–i). The overexpression of circRIMS2 was found to decrease the protein levels of synaptic proteins, including GluN2B and GluN2A (Fig. 3j). Moreover, circRIMS2 was observed to sponge miR-3968 and lead to a reduction of miR-3968 in vivo (Fig. 3k). These findings suggest that the overexpression of circRIMS2 induces memory and synaptic impairments in vivo.

### circRIMS2 promotes UBE2K/GluN2B ubiquitination by sponging miR-3968

**Fig. 4:**
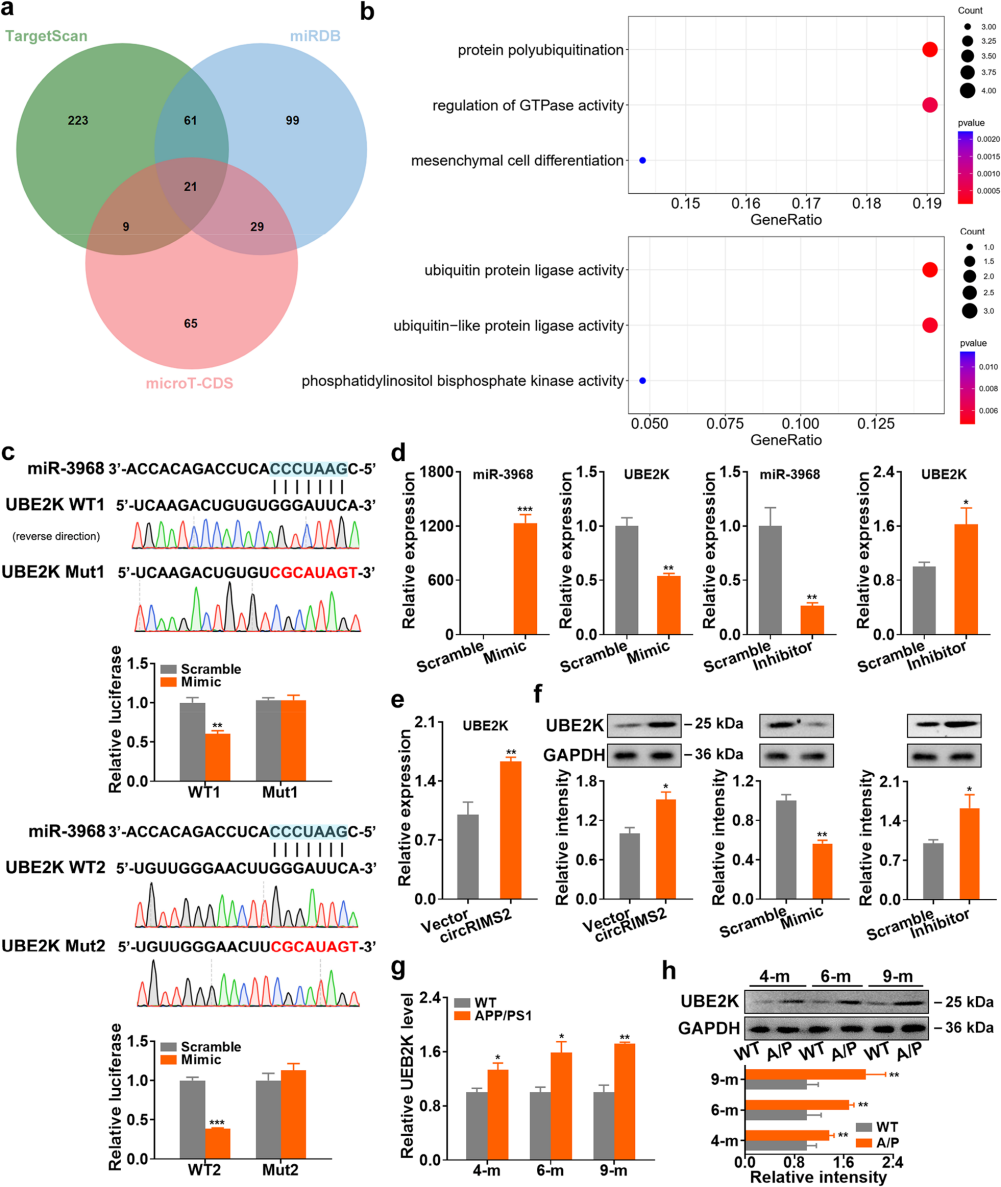
UBE2K is a target of miR-3968. **a** Venn diagram illustrates the intersection of predicted target genes of miR-3968 from three distinct online tools. **b** The biological processes (BP) and molecular function (MF) GO terms for the 21 intersected genes (depicted in **a**) were predicted by clusterProfiler R package (Version 4.8.3). **c** Two different predicted sites of miR-3968 binding within the 3′-UTR of UBE2K and two distinct mutant sequences of the UBE2K 3′-UTR were constructed. Dual-luciferase reporter assays revealed that miR-3968 mimic could only suppress the luciferase activity of WT 3′-UTR of UBE2K in HEK293 cells (*n* = 3). **d** The mRNA levels of miR-3968 and UBE2K were detected by qRT-PCR in N2a cells transfected with miR-3968 mimic (Mimic), inhibitor (Inhibitor), or the corresponding scramble control (Scramble) (*n* = 4). **e** The mRNA level of UBE2K in N2a cells transfected with circRIMS2 (*n* = 4). **f** The protein levels of UBE2K in N2a cells with transfection of Mimic or Inhibitor were detected by WB (*n* = 3). **g** qRT-PCR analysis of UBE2K in the hippocampus of APP/PS1 and WT mice at diverse stages (*n* = 4). **h** The protein levels of UBE2K in the hippocampus of WT and APP/PS1 mice at different stages were detected by WB (*n* = 4). Data are presented as mean ± S.E.M. and two-tailed *t* tests were used unless otherwise specified. *P < 0.05, **P < 0.01, ***P < 0.001

TargetScan v7.2 [23], miRDB [24], and microT-CDS [25] were concurrently used to predict the targets of miR-3968, which mediates synaptic dysfunction in AD (Additional file 1: Table S5). Twenty-one genes were revealed by all three softwares, which were enriched in protein ubiquitination GO terms as analyzed by the clusterProfiler (v4.6.2) R package [26] (Fig. 4a, b). Based on this analysis, we selected TRIM62, RC3H1, and ubiquitin conjugating enzyme E2 K (UBE2K) as candidates for further validation, as they were enriched in both GO biological processes and molecular functions associated with protein ubiquitination. Our subsequent experiments showed that only the protein level of UBE2K was significantly downregulated in miR-3968-overexpressing N2a cells (Additional file 1: Fig. S3), indicating that UBE2K is a potential target of miR-3968. To further confirm this finding, we performed a luciferase activity assay and found that miR-3968 only reduced the luciferase activity of WT UBE2K but not the two mutants of UBE2K 3′UTR in HEK293 cells (Fig. 4c). Expression of UBE2K was decreased and increased in N2a cells transfected with miR-3968 mimic and inhibitor, respectively, while circRIMS2 had the opposite effect (Fig. 4d–f). Moreover, UBE2K was gradually increased in APP/PS1 mice from 4 months (Fig. 4g, h), which was consistent with the change in circRIMS2. Finally, we observed that upregulation of miR-3968 or silencing UBE2K could rescue memory and synaptic impairments in mice overexpressing circRIMS2 (Additional file 1: Fig. S4).

**Fig. 5:**
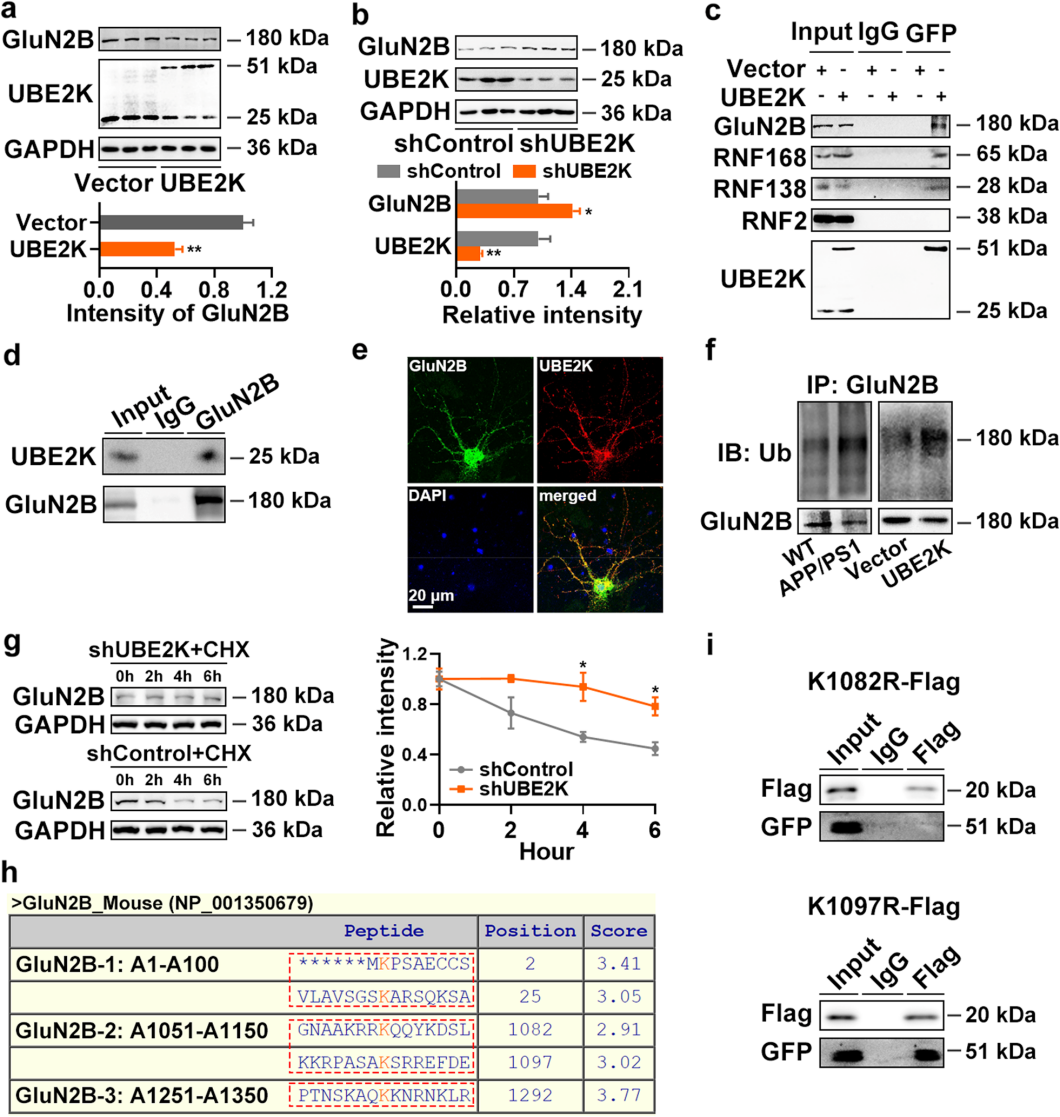
UBE2K binds directly to GluN2B and facilitates its ubiquitination. **a** Protein levels of GluN2B and UBE2K in N2a cells overexpressing UBE2K were examined by WB (*n* = 3). **b** Protein levels of GluN2B and UBE2K in N2a cells transfected with UBE2K shRNA (shUBE2K) or control (shControl) for 72 h (*n* = 3). **c** Immunoprecipitation was performed using 500 μg of proteins from cell lysates transfected with vector or UBE2K-GFP fused plasmid, using nonspecific IgG (IgG) and anti-GFP antibodies. The resulting precipitates were blotted with antibodies against GluN2B, RNF168, RNF138, RNF2, or UBE2K. Additionally, 30 μg of protein from the extracts without immunoprecipitation were loaded as input. **d** Immunoprecipitation was performed using 500 μg of proteins extracted from the hippocampus of 4-month-old C57BL/6 mice, using IgG and anti-GluN2B antibodies. The resulting precipitates were blotted with antibodies against GluN2B and UBE2K. **e** Cultured mouse cortical neurons at DIV14 were stained with antibodies against GluN2B (green) and UBE2K (red), as well as DAPI (blue). Scale bar, 20 μm. **f** An ubiquitination assay was performed to analyze GluN2B ubiquitination in the hippocampus of 4-month-old WT and APP/PS1 mice, as well as in N2a cells transfected with UBE2K for 48 h. **g** WB was performed to assess the effect of shUBE2K on the protein degradation rate of GluN2B in the presence of CHX (*n* = 3). **h** Using GPS-Uber (http://gpsuber.biocuckoo.cn/online.php), five ubiquitination sites (K2, K25, K1082, K1097, and K1293) on GluN2B were predicted and highlighted on the peptide sequence. Subsequently, we generated three fragments of GluN2B, each containing one of these ubiquitination sites. **i** N2a cells were co-transfected with UBE2K-GFP and WT or K1082R/K1097R GluN2B-2 fragment for 48 h. Proteins were pulled down using IgG and anti-Flag antibodies, and further detected by WB using antibodies against GluN2B-2 (Flag) and UBE2K (GFP). Data are presented as mean ± SEM and two-tailed* t* tests were used unless otherwise specified. *P < 0.05, **P < 0.01

We further investigated the downstream targets of UBE2K in mediating synaptic impairment in AD, and found that GluN2B, a subunit of *N*-methyl-*D*-aspartate (NMDA) receptor essential for neuronal communication [27], may be its target. Overexpression of circRIMS2 led to a substantial reduction of GluN2B protein level, while upregulation of miR-3968 or silencing of UBE2K reversed this reduction (Fig. 3j; Additional file 1: Fig. S4j, k). Our findings suggest that UBE2K may directly interact with GluN2B, mediating its ubiquitination and degradation, as overexpression of UBE2K decreased GluN2B protein levels while silencing of UBE2K increased them (Fig. 5a, b). N2a cells were transfected with the UBE2K-GFP construct, and Co-IP identified that UBE2K interacted with GluN2B, RING finger family 168 (RNF168), and RNF138 but not RNF2 (Fig. 5c). RNF138 and RNF2 are the main E3 enzymes interacting with UBE2K [28]. Meanwhile, UBE2K could also be pulled down by GluN2B antibody in C57BL/6 mice (Fig. 5d). Immunofluorescence experiments revealed colocalization of UBE2K and GluN2B in primary cortical mouse neurons (Fig. 5e). Moreover, the protein level of GluN2B was decreased while the ubiquitination level was increased in APP/PS1 mice and UBE2K-transfected N2a cells (Fig. 5f). In addition, CHX, a popular inhibitor of protein synthesis, was used to assess the effect of UBE2K on GluN2B protein stability, and we found that silencing of UBE2K slowed down the degradation of GluN2B considerably (Fig. 5g). We used GPS-Uber [29] (http://gpsuber.biocuckoo.cn/) to predict the potential ubiquitination sites within GluN2B, and found five highly conserved ubiquitination sites (Fig. 5h). We generated three fragments of GluN2B containing these ubiquitination sites and found that GluN2B-2 containing GluN2B K1082 and K1097 could be successfully pulled down by UBE2K in N2a cells, while K1082R but not K1097R mutation abolished this interaction (Fig. 5i, Additional file 1: Fig. S5a, b). We also explored whether miR-3968 could target GluN2B directly. Three poorly conserved potential miR-3968-binding sites were predicted within the 3′UTR of GluN2B by TargetScan (Additional file 1: Fig. S5c–e); however, the luciferase activity assay revealed no suppressive effects of miR-3968 on these three sites (Additional file 1: Fig. S5f), suggesting that GluN2B might not be the direct target of miR-3968. These data suggest that upregulation of the circRIMS2/miR-3968 pathway may result in aberrant UBE2K activation and GluN2B degradation via ubiquitination of GluN2B at the K1082 site.

### Overexpression of miR-3968 or silencing UBE2K rescues synaptic and memory impairments in APP/PS1 mice

**Fig. 6:**
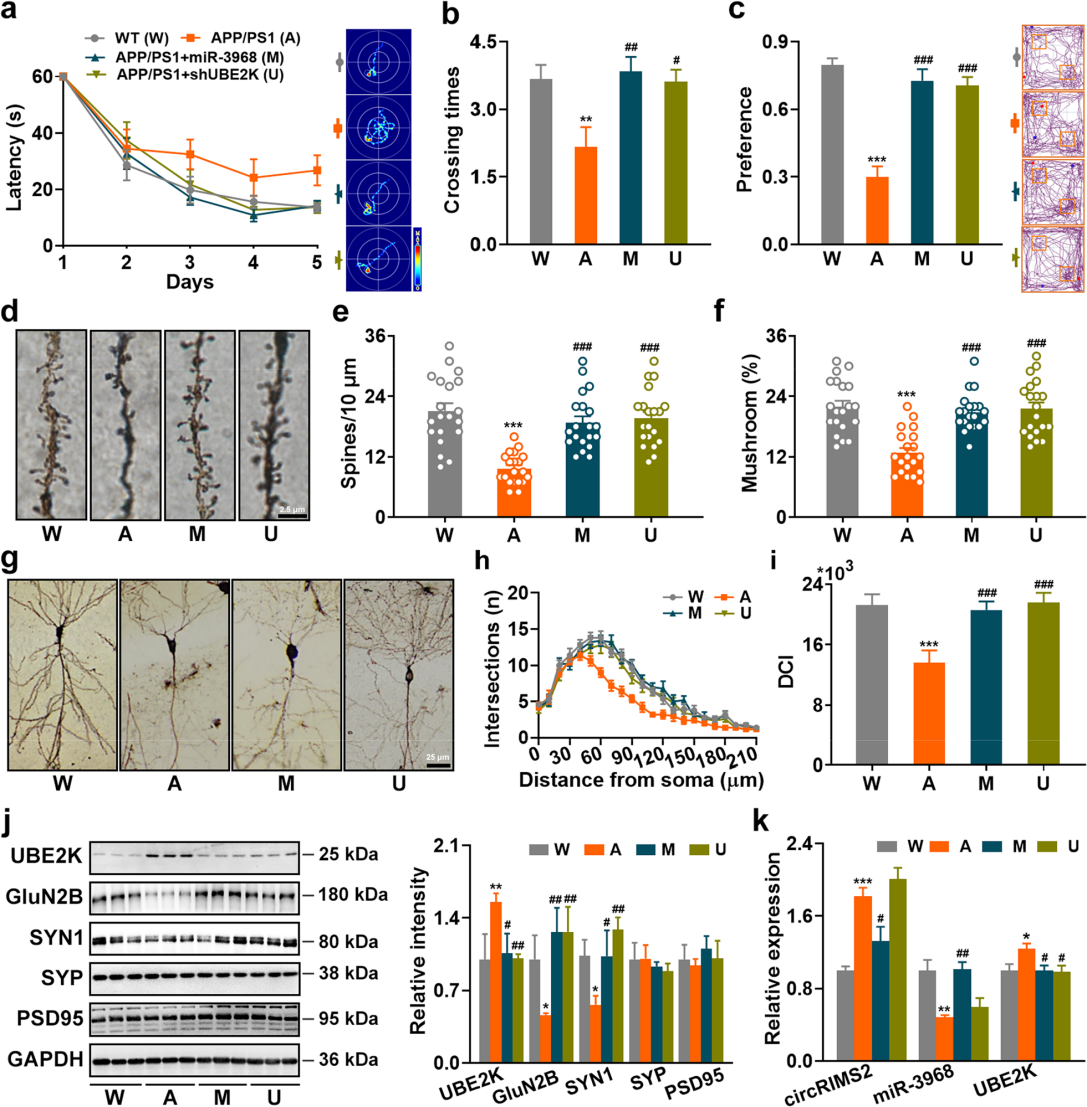
Overexpression of miR-3968 or silencing UBE2K rescues synaptic and memory impairments in AD mice. Lentivirus containing miR-3968 or shUBE2K was injected into the bilateral hippocampi of 5-month-old APP/PS1 mice. Four weeks later, the mice underwent NOR and MWM, and then were sacrificed for WB and Golgi staining. The experimental groups were as follows: W—WT mice, A—APP/PS1 mice, M—APP/PS1 mice injected with miR-3968 lentivirus, U—APP/PS1 mice injected with shUBE2K lentivirus. **a, b** Performance in the MWM test. Latency during learning stage was recorded (**a**). The representative traces (**a**) and crossing times (**b**) on day 7 were analyzed. The study was conducted on 15 WT mice, 12 APP/PS1 mice, 13 APP/PS1 mice injected with miR-3968 lentivirus, and 13 APP/PS1 mice injected with shUBE2K lentivirus. Data were analyzed with one-way ANOVA with LSD *post-hoc* test. **c** Recognition memory was tested using the NOR. Data were analyzed with one-way ANOVA with LSD *post-hoc* test. **d–f** Golgi-cox staining was performed to demonstrate spine density and maturation. **d** Representative images of dendritic spines. Scale bar, 2.5 μm. **e** Quantitative analysis of spine density and **f** the percentage of mushroom-type spines are presented. The data were obtained from 20 neurons per group (one-way ANOVA with Tukey’s *post-hoc* test). **g–i** Dendritic morphology of neurons was analyzed using Golgi staining. **g** Representative images of dendritic trees are shown, with a scale bar of 25 μm. Sholl analysis (**h**) and DCI analysis (**i**) were performed to evaluate dendritic complexity. **j** Protein levels of UBE2K, GluN2B, SYN1, SYP, and PSD95 were measured in hippocampal homogenates from four different groups using WB (left). Quantitative analysis was performed on data obtained from three samples in each group (right). **k** qRT-PCR analysis was performed to detect circRIMS2, miR-3968, and UBE2K in hippocampal homogenates from four different groups (*n* = 4). Data are presented as mean ± SEM and two-tailed* t* tests were used unless otherwise specified. *P < 0.05, **P < 0.01, ***P < 0.001 vs W; ^#^*P* < 0.05, ^##^*P* < 0.01, ^###^*P* < 0.001 vs A

We also investigated whether downregulation of the circRIMS2/miR-3968/UBE2K pathway could rescue the synaptic and memory impairments in AD. Silencing of circRIMS2 was difficult due to the consecutive six A bases around the back-splicing site of circRIMS2 (Fig. 1g), so we downregulated the pathway via upregulation of miR-3968 or silencing UBE2K. Injection of lentivirus containing miR-3968 or shUBE2K into the hippocampus of APP/PS1 mice partially rescued memory impairments in both MWM and NOR tests (Fig. 6a–c, Additional file 1: Fig. S6), restored the density, maturation, and complexity of neuronal dendrites (Fig. 6d–i), and reversed the protein levels of synaptic proteins in APP/PS1 mice (Fig. 6j, k), suggesting that downregulation of the circRIMS2/miR-3968/UBE2K pathway remarkably rescued synaptic and memory impairments in AD.

### Blocking UBE2K/GluN2B ubiquitination by a short peptide rescues synaptic and memory impairments in APP/PS1 mice

**Fig. 7:**
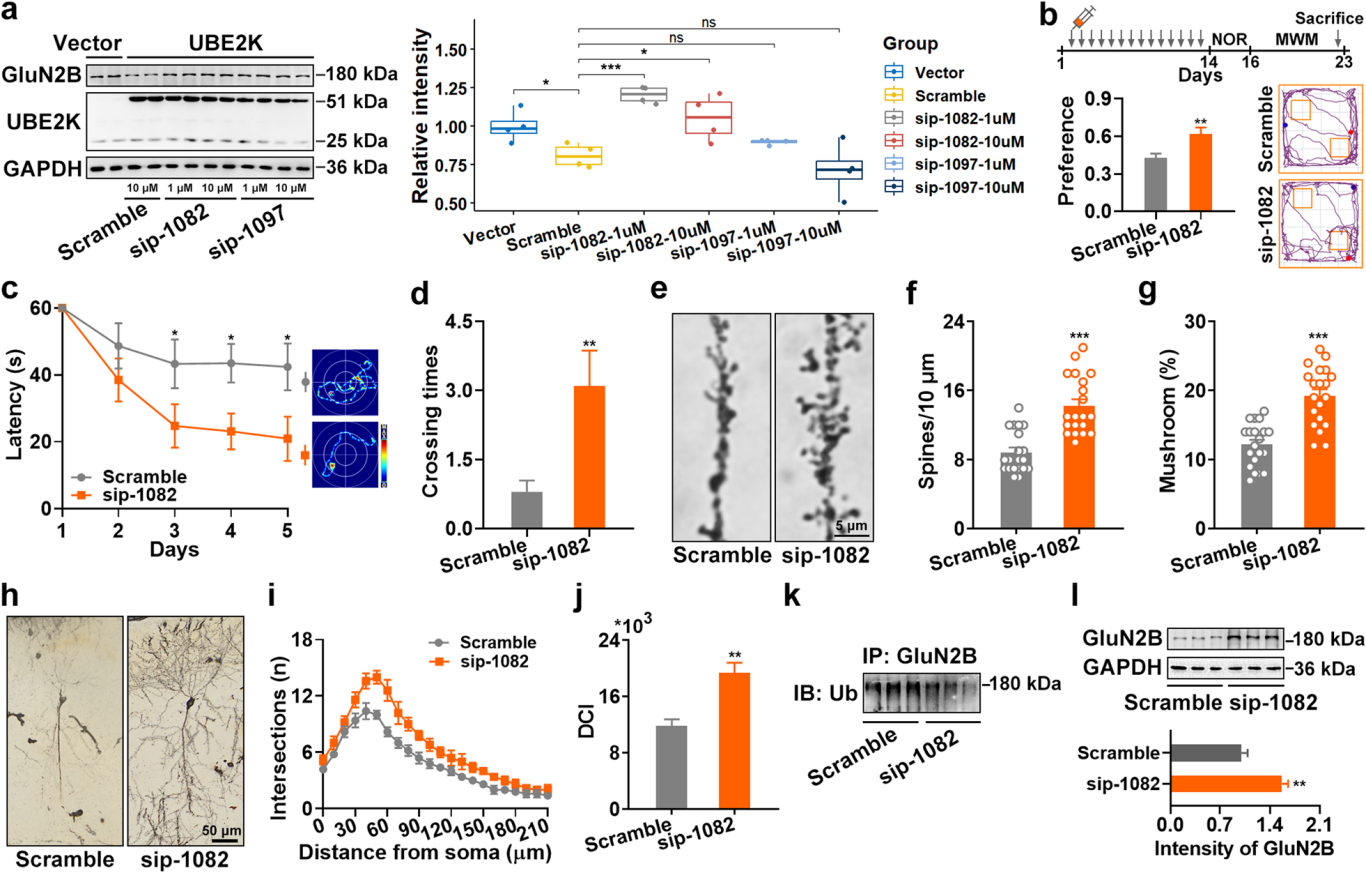
A peptide blocking UBE2K/GluN2B ubiquitination rescues synaptic and memory impairments in APP/PS1 mice. **a** N2a cells were transfected with UBE2K for 12 h, followed by treatment with sip-1082 or sip-1097 at 1 μM or 10 μM for an additional 24 h, as indicated. The protein levels of GluN2B and UBE2K were tested by WB (*n* = 4). **b** Upper part: A schematic diagram of the experiment is presented. The sip-1082 or scramble peptide (15 mg/kg per day) was injected intraperitoneally in 12-month-old APP/PS1 mice for a period of 2 weeks. Then, the mice underwent NOR and MWM, as well as WB and Golgi staining. Lower part: The preference index of the NOR test was calculated. **c, d** The performance in MWM, including the latencies and the representative traces during the learning stage (**c**), and the crossings on day 7 (**d**) (*n* = 10). **e–g** Golgi-cox staining was performed to assess the dendritic spines, and representative images are presented (**e**). Changes of spine density (**f**) and the percentage of mushroom spines (**g**) are presented. **h–j** The representative images of the dendritic trees (**h**). Scale bar, 50 μm. The Sholl analysis evaluates the distribution of dendritic intersections (**i**). The DCI analysis assesses the overall complexity of the dendritic arbor (**j**). **k, l** Co-Ip (**k**) and WB (**l**) to examine the ubiquitination and protein levels of GluN2B in hippocampus of APP/PS1 mice injected with sip-1082 or scramble peptides (*n* = 3). Data are presented as mean ± SEM and two-tailed* t* tests were used unless otherwise specified. *P < 0.05, **P < 0.01, ***P < 0.001

We examined whether blocking the ubiquitination of GluN2B could rescue synaptic and memory impairments in AD. Drawing from our previous experience [18], we generated two peptides that overlapped the K1082 and K1097 ubiquitination sites, respectively. Our results indicated that sip-1082 (around K1082), but not sip-1097 (around K1097), could reverse the decrease of GluN2B protein level caused by UBE2K overexpression in N2a cells (Fig. 7a). These findings were consistent with the Co-IP results, which showed that only K1082R but not K1097R mutation abolished the interaction between UBE2K and GluN2B-2 (Fig. 5i). We further administered sip-1082 (15 mg/kg, i.p.) to 12-month-old APP/PS1 mice for 14 consecutive days. The treatment significantly improved their learning performance in both NOR and MWM tests (Fig. 7b–d), restored the density, maturation, and complexity of neuronal dendrites (Fig. 7e–j), suppressed GluN2B ubiquitination (Fig. 7k), and increased GluN2B protein level (Fig. 7l). Our results indicate that blocking the UBE2K-mediated ubiquitination of GluN2B remarkably rescues synaptic and memory impairments in AD mice.

### Silencing METTL3 mitigates AD pathology in APP/PS1 mice

**Fig. 8:**
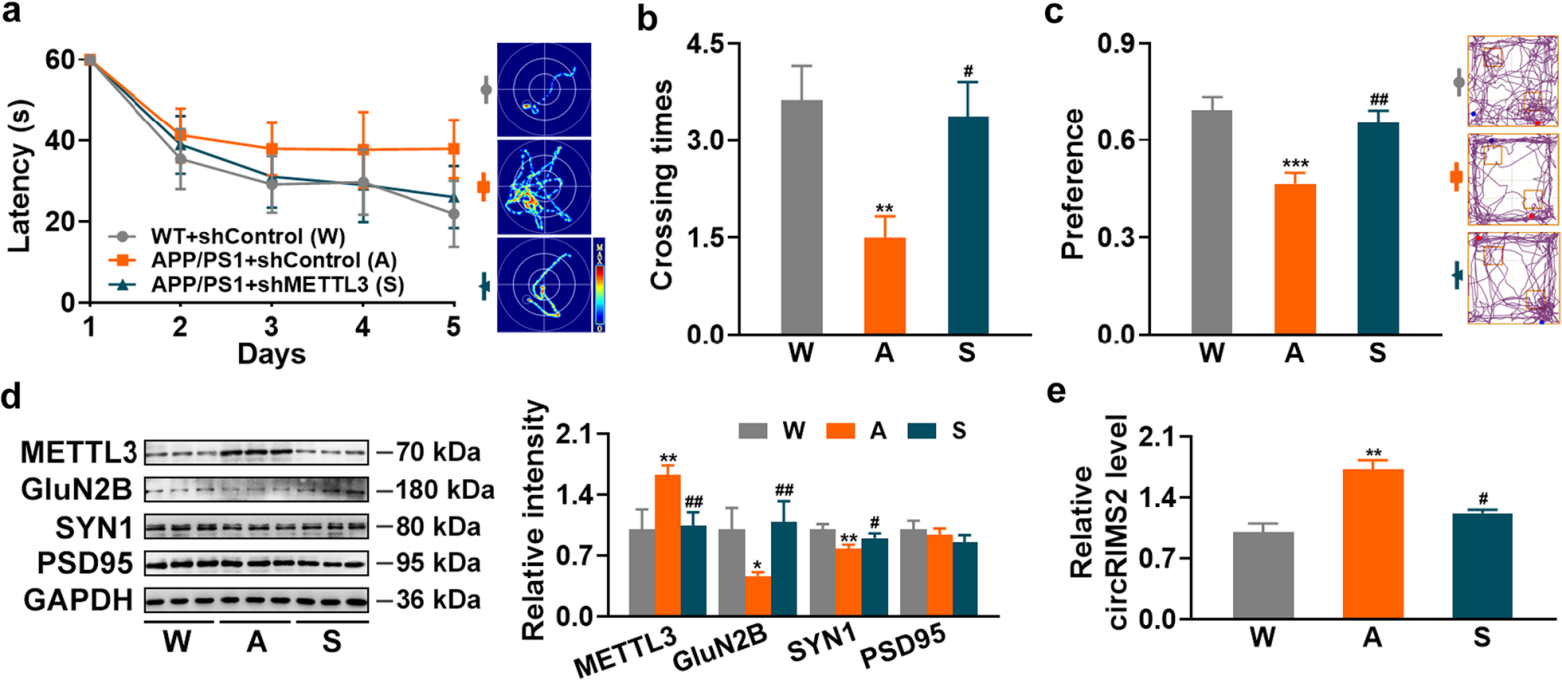
Silencing METTL3 mitigates AD pathology in APP/PS1 mice. AAV containing shMETTL3 or control (shControl) was injected into the bilateral hippocampi of 5-month-old APP/PS1 mice. Two weeks later, the mice underwent NOR and MWM, and were sacrificed for WB and qRT-PCR analyses. **a, b** Performance in MWM. Latency during the learning stage was recorded (**a**). The representative traces (**a**) and crossing times (**b**) on day 7 were analyzed (*n* = 8). The experimental groups were as follows: W—WT mice injected with shControl AAV, A—APP/PS1 mice injected with shControl AAV, S—APP/PS1 mice injected with shMETTL3 AAV. Statistical analysis was performed using one-way ANOVA with LSD *post-hoc* test for (**b**). **c** Recognition memory was tested by NOR. Statistical analysis was performed using one-way ANOVA with LSD *post-hoc* test. **d** WB for METTL3, GluN2B, SYN1, and PSD95 in hippocampal homogenates (left) and quantitative analysis (right) (*n* = 3). **e** qRT-PCR analysis was performed to detect circRIMS2 (*n* = 3). Data are presented as mean ± SEM and two-tailed *t* tests were used unless otherwise specified. *P < 0.05, **P < 0.01 vs W; ^#^*P* < 0.05, ^##^*P* < 0.01 vs A

The results above revealed that blocking the circRIMS2/miR-3968/UBE2K/GluN2B axis remarkably ameliorated the synaptic dysfunction in APP/PS1 mice. We also investigated whether silencing METTL3, the upstream regulator of circRIMS2, could mitigate AD pathology in APP/PS1 mice. MeRIP-PCR was applied to detect the m6A levels of UBE2K and GluN2B after transfection of METTL3 in N2a cells, and the results showed that the m6A levels of UBE2K and GluN2B were not affected by METTL3 (Additional file 1: Fig. S7), partially excluding the direct m6A modification regulation of UBE2K and GluN2B by METTL3. Then 5-month-old APP/PS1 mice were injected with shMETTL3 or control AAV, and behavioral tests were performed two weeks later. Silencing METTL3 partially rescued memory impairments in both MWM and NOR tests (Fig. 8a–c), reversed the protein levels of synaptic proteins as well as expression and m6A levels of circRIMS2 in APP/PS1 mice (Fig. 8d, e, Additional file 1: Fig. S8), suggesting that downregulation of METTL3 remarkably mitigated AD pathology in APP/PS1 mice.

## Discussion

**Fig. 9:**
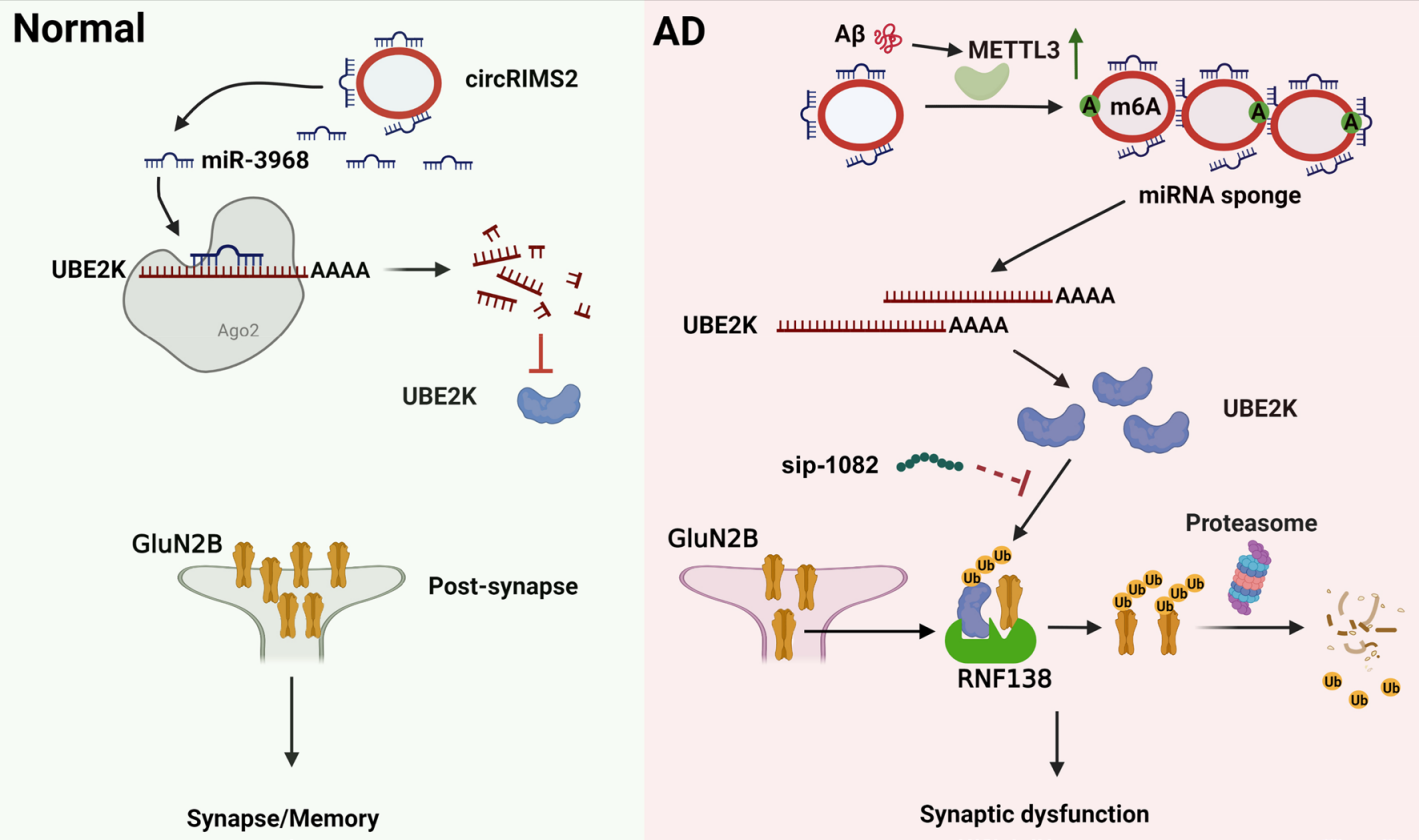
The dysfunction of circRIMS2/miR-3968/UBE2K/GluN2B results in synaptic and memory impairments in AD mice. Under normal condition (left), miR-3968 inhibits UBE2K, and the protein level of GluN2B is maintained. However, in AD (right), Aβ induces an elevation in METTL3, which enhances the stability of circRIMS2 through m6A modification. Consequently, increased circRIMS2 binds competitively with miR-3968, leading to the overexpression of UBE2K. UBE2K interacts with GluN2B, mediating its ubiquitination, degradation, and synaptic dysfunction. The partial rescue of these abnormalities could be achieved by blocking the ubiquitination of K1082 on GluN2B using sip-1082 peptide

In the present study, we identified an abnormally upregulated circRIMS2/miR-3968 ceRNA pair in AD. The upregulation of this pathway resulted in increased UBE2K and ubiquitination-mediated degradation of GluN2B, leading to memory and synaptic impairments. Moreover, blocking the circRIMS2/miR-3968/UBE2K/GluN2B axis remarkably ameliorated the synaptic dysfunction in APP/PS1 mice (Fig. 9).

Although circRNAs are enriched in neuronal tissues, their contribution to neurodegenerative diseases such as AD remains poorly understood [6]. Some previous studies have reported dysregulation of circRNAs in pre-symptomatic AD, including downregulated circHOMER1, which is negatively linked with the Clinical Dementia Rating and Braak score in AD patients [5]. Other studies have characterized the circRNA-miRNA ceRNA networks and identified variations in circRNA expression between brain regions in AD and related dementias [8, 9]. Additionally, Trinchese et al. [17] summarized age-related progression in APP/PS1 mice, and observed that both long-term potentiation and short-term memory are impaired in 3-month-old APP/PS1 mice, and become worsened as the mice are getting older, indicating that synaptic impairments occur at an early stage before obvious Aβ deposits are formed. Also, we have observed dendritic spine impairment in 4-month-old APP/PS1 mice [30], which prompted us to characterize the ceRNA network changes in early-stage APP/PS1 mice and identify 11 circRNA/miRNA ceRNA pathways among differentially expressed circRNAs and miRNAs. Ultimately, we identified an upregulated circRIMS2/miR-3968 ceRNA network where circRIMS2 was upregulated and miR-3968 was downregulated. This change was consistent among APP/PS1 mice, 3 × Tg mice and Aβ-treated primary mouse cortical neurons. Moreover, overexpression of circRIMS2 in 4-month-old C57BL/6 mice induced AD-like memory and synaptic impairments.

The m6A modification is linked to the whole life cycles of RNAs [31], and may promote the degradation or increase the stability of some circRNAs. For example, circQSOX1 could be m6A modified by METTL3, and further read by IGF2BP2, and knockdown of METTL3 or IGF2BP2 significantly decreased circQSOX1 [32]. Here, we found that the m6A level of circRIMS2 was upregulated in 4-month-old APP/PS1 mice. The m6A writer (METTL3) and readers (YTHDC1, IGF2BP1) were also upregulated. YTHDC1, which regulates mRNA alternative splicing [33], is upregulated in the brains of AD patients, and positively correlated with APP transcripts [34]. IGF2BP1 was found to stabilize target mRNAs in an m6A-dependent manner [35]. Dysregulation of METTL3-dependent m6A in AD has been widely reported with controversial results. Significant upregulation of METTL3 protein in the insoluble fractions has been observed in the hippocampus of AD patients, which positively correlates with insoluble tau protein levels [36]. Zhao et al. observed elevated m6A immunoreactivity in astrocytes and microglia and reduced m6A immunoreactivity in neurons in AD patient brains. However, no significant difference of total m6A level was found between AD and control brains, partially due to the opposite changes in neurons and glial cells. Moreover, overexpression of METTL3 ameliorated synaptic and cognitive impairments in rats treated with Aβ [37]. Han et al. [38] also identified upregulated METTL3 and downregulated FTO in 9-month-old APP/PS1 mice. Recently, Yin et al. [39] reported that loss of METTL3 in macrophages decreased the m6A modification of DNA methyltransferase 3A, reduced the expression of alpha-tubulin acetyltransferase 1, decreased the acetylation of α-tubulin, and improved Aβ clearance by macrophages. Tang et al. [40] reported that KDM1A-mediated METTL3 overexpression promoted autophagic clearance of p-Tau via stabilizing STUB1 mRNA in an IGF2BP1-dependent manner. Our study utilized a combination of SRAMP online prediction, MeRIP-PCR, RAP, and mass spectrometry analysis to identify that METTL3 increases the m6A level of circRIMS2 by stabilizing it. Moreover, silencing METTL3 by sh-METTL3 AAV ameliorated synaptic and cognitive impairments in 6-month-old APP/PS1 mice. Temporal-, spatial-, and tissue-specific dynamics of m6A modification occurs during aging [41]. The controversy regarding how METTL3-m6A changes in AD patients may be partially caused by the heterogeneity of patients including sex ratio, Braak stage, and *APOE4* allele status. In animal models, studies by Han et al. [38] and our study both showed upregulation of METTL3 in APP/PS1 mice, and beneficial effects of silencing METTL3 in 6-month-old APP/PS1 mice and Aβ-induced mice [39]. However, opposite results were observed in Aβ-induced rats [37]. Tang et al. [40] reported beneficial effects of METTL3 upregulation in APP/PS1 mice; however, the age of mice was not clear. These results indicate the complicated involvement of METTL3 and m6A modification in AD by targeting different downstream targets and pathways under different conditions. Further investigation is needed to determine the detailed involvement and interaction among METTL3, IGF2BP1, and circRIMS2. These findings highlight the potential therapeutic applications of the m6A system for AD.

In maintaining neuronal morphology and synaptic plasticity, miRNA has been identified as a common target of circRNA or lncRNA, with several miRNAs playing crucial roles in mediating the synaptic dysfunction observed in AD [18, 42]. Our study discovered that miR-3968 was a downstream target of circRIMS2 and was downregulated in AD models. Overexpression of miR-3968 in APP/PS1 mice promoted spine maturation, dendritic complexity, and learning. Huang et al. [43] also reported decrease of miR-3968 in the hippocampus of mice with chronic social defeat stress, with overexpression of miR-3968 rescuing depression-like behaviors. As depression is a risk factor and early symptom of AD, with up to 40% of AD patients experiencing significant depression [1], administration of miR-3968 may be effective for both emotional and cognitive disorders in AD.

To predict the targets of miR-3968, three online algorithms were employed in this study, and the intersected targets were found enriched in the protein ubiquitination pathway. UBE2K was subsequently confirmed as the direct target of miR-3968 in AD. UBE2K belongs to the E2 ubiquitin conjugating enzyme family and is widely expressed and enriched in the brain [44]. E2 ubiquitin conjugating enzymes play critical roles in maintaining synaptic function. Mutations of *UBE2A* lead to reduced synaptic transmission due to mitochondrial failure in *Drosophila* [45]. Knockout of *UBE2A* impairs hippocampal learning and memory in mice [46]. UBCH8 interacts with Parkin to mediate the ubiquitination of CDCrel-1, a synaptic vesicle protein [47]. Increased protein levels of UBE2K have also been observed in both AD patients and Tg2576 transgenic mice [48]. UBE2K mediates Aβ neurotoxicity by inhibiting proteasome activity, promoting apoptosis accompanied by proteolytic activation of caspase-12 triggered by endoplasmic reticulum [49]. Nevertheless, the role of UBE2K in synaptic function remains poorly understood.

Posttranslational modifications (PTMs) significantly alter the functions of proteins, and disruption in their regulation could lead to abnormal pathology. PTMs characterize some of the most discussed players in AD, including Aβ and tau, through processes such as phosphorylation, ubiquitination, acetylation, and glycosylation [50]. We previously identified that miR-124 downregulated the expression of PTPN1, and promoted the phosphorylation of GluA2 on Y876 as well as internalization of GluA2, resulting in the loss of membranous GluA2 in AD [18]. Loss of GluN2B in CA1 hippocampus impaires dendritic spine density and hippocampal-mediated learning and memory, indicating the essential role of GluN2B in synaptic plasticity regulation [51]. In this study, GluN2B was substantially decreased in both APP/PS1 mice and mice overexpressing circRIMS2. We further validated that UBE2K interacted with GluN2B and mediated its ubiquitination and degradation involving its K1082 ubiquitination site. Moreover, disruption of the circRIMS2/miR-3968/UBE2K/GluN2B pathway robustly rescued the synaptic and learning impairments in APP/PS1 mice.

Although short peptides linked with TAT have been widely used to block PTM (especially phosphorylation) of synaptic proteins [42, 52], the application of peptides for blocking ubiquitination modification of synaptic proteins is missing. Our study found that the short peptide sip-1082 specifically blocked the interaction between UBE2K and GluN2B, decreased the ubiquitination of GluN2B, and increased its protein level, resulting in improved learning and memory in 12-month-old APP/PS1 mice. These data indicate a novel therapeutic approach for AD.

## Conclusions

In summary, we characterized the circRNA/miRNA expression pattern at an early stage of AD in which the mice had synaptic dysfunction but without obvious Aβ deposition. We revealed an essential role of the upregulated circRIMS2/miR-3968 pathway in mediating synaptic and memory impairments in AD through activating the UBE2K-mediated ubiquitination and degradation of GluN2B. Moreover, we revealed that blocking the ubiquitination of GluN2B could ameliorate the synaptic dysfunction in AD model mice. Our work proposes several novel therapeutic targets for overcoming synaptic and memory impairments in AD.

### Supplementary Information

**Additional file 1: Fig. S1**. circRIMS2 functions as a miRNA sponge of miR-3968. **Fig. S2.** METTL3 mediated m6A modification of circRIMS2. **Fig. S3.** Downstream target validation of miR-3968. **Fig. S4.** Overexpression of miR-3968 or silencing UBE2K rescues circRIMS2 induced memory impairment and synaptic disorders in vivo. **Fig. S5.** GluN2B-2 interacted with UBE2K. **Fig. S6.** Injection of control lentivirus did not affect the learning and memory of WT mice. **Fig. S7.** METTL3 did not affect the m6A modification of UBE2K and GluN2B. **Fig. S8.** Silencing METTL3 reversed the m6A level of circRIMS2 in APP/PS1 mice. **Table S1.** List of the primary and secondary antibodies. **Table S2.** The dysregulated circRNAs in the hippocampus of 4-month-old APP/PS1 mice. **Table S3.** The dysregulated miRNAs in the hippocampus of 4-month-old APP/PS1 mice. **Table S4.** The predicted circRNA/miRNA ceRNA pairs by miRanda. **Table S5.** The predicted targets of miR-3968.

## Data Availability

The datasets used and/or analysed during the current study are available in the GEO repository, GSE166393 (https://www.ncbi.nlm.nih.gov/geo/query/acc.cgi?acc=GSE166393).

## Abbreviations

AD: Alzheimer's disease
WT: Wild type
Aβ: Amyloid-β
circRNA: Circular RNA
ceRNA: Competing endogenous RNA
METTL3: Methyltransferase 3, N6-adenosine-methyltransferase complex catalytic subunit
m6A: N6-methyladenosine
UBE2K: Ubiquitin conjugating enzyme E2 K
RIMS2: Regulating synaptic membrane exocytosis 2
ActD: Actinomycin D
