# Supplementary material for: N6-methyladenosine-modified circRIMS2 mediates synaptic and memory impairments by activating GluN2B ubiquitination in Alzheimer's disease
Source: Transl Neurodegener. 2023 Nov 28;12:53. doi: 10.1186/s40035-023-00386-6 (PMC10683276; doi:10.1186/s40035-023-00386-6)
Supplement: Supplementary file 1 — Additional file 1: Fig. S1. circRIMS2 functions as a miRNA sponge of miR-3968. Fig. S2. METTL3 mediated m6A modification of circRIMS2. Fig. S3. Downstream target validation of miR-3968. Fig. S4. Overexpression of miR-3968 or silencing UBE2K rescues circRIMS2 induced memory impairment and synaptic disorders in vivo. Fig. S5. GluN2B-2 interacted with UBE2K. Fig. S6. Injection of control lentivirus did not affect the learning and memory of WT mice. Fig. S7. METTL3 did not affect the m6A modification of UBE2K and GluN2B. Fig. S8. Silencing METTL3 reversed the m6A level of circRIMS2 in APP/PS1 mice. Table S1. List of the primary and secondary antibodies. Table S2. The dysregulated circRNAs in the hippocampus of 4-month-old APP/PS1 mice. Table S3. The dysregulated miRNAs in the hippocampus of 4-month-old APP/PS1 mice. Table S4. The predicted circRNA/miRNA ceRNA pairs by miRanda. Table S5. The predicted targets of miR-3968. [file 40035_2023_386_MOESM1_ESM.docx]

**Supplementary Information**

**N6-methyladenosine-modified circRIMS2 mediates synaptic and memory impairments by activating GluN2B ubiquitination in Alzheimer's disease**

Xiong Wang, Jiazhao Xie, Lu Tan, Yanjun Lu, Na Shen, Jiaoyuan Li, Hui Hu, Huijun Li, Xiaoguang Li, Liming Cheng

**SupplementaryFigures and Figure legend**

**
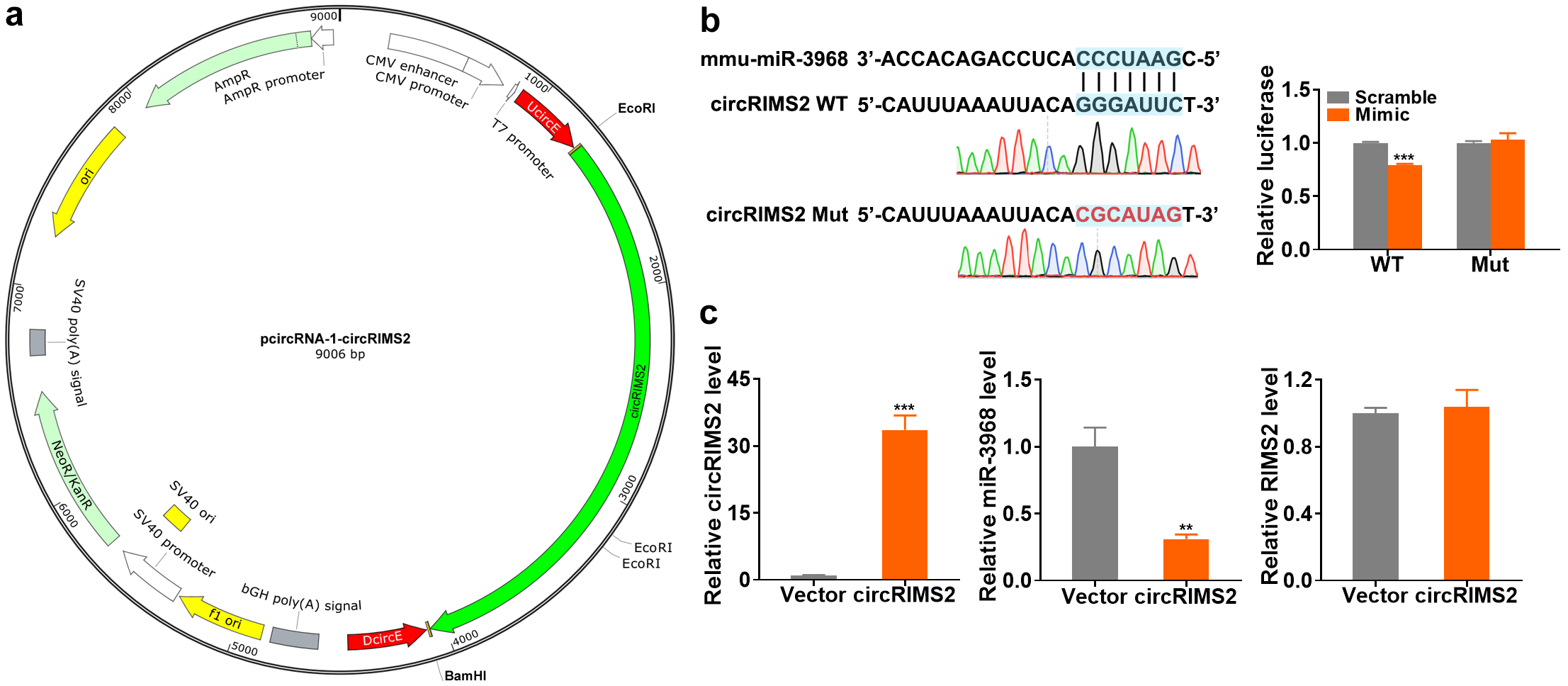
**

**Fig. S1 circRIMS2 functions as a miRNA sponge of miR-3968. a** The whole circRIMS2 sequence was cloned into pcircRNA-1 circRNA overexpression vector purchased from BersinBio (Guangzhou, China). **b** We constructed the predicted binding site of miR-3968 within the circRIMS2 sequence (WT), along with a distinct mutant sequence of circRIMS2 (Mut). Dual-luciferase reporter assays conducted in HEK293 cells (*n =* 3) demonstrated that the miR-3968 mimic reduced the luciferase activity of WT circRIMS2 only. **c** Expression of circRIMS2, miR-3968, and RIMS2 was analyzed through qRT-PCR in N2a cells transfected with vector or circRIMS2 for 48 hours (*n =* 4). (Data are presented as mean ± S.E.M. and two-tailed t tests were used unless otherwise specified. Source data are provided as a Source Data file. ***P* < 0.01, ****P*< 0.001.)


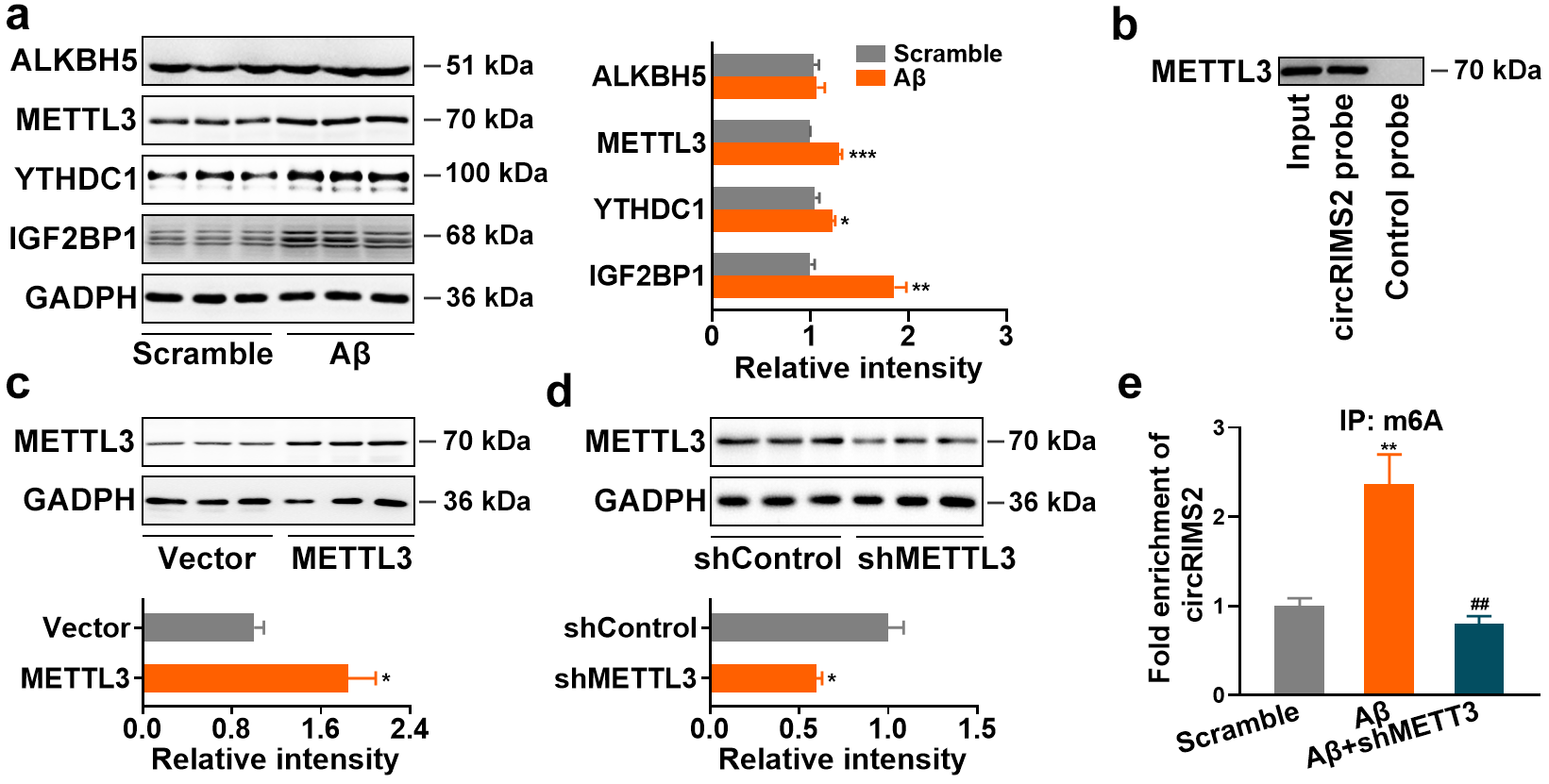


**Fig. S2 METTL3 mediated m6A modification of circRIMS2. a** The protein levels of ALKBH5, METTL3, YTHDC1, and IGF2BP1 were quantified by WB in mouse primary cortical neurons treated with Aβ or scramble (left). The results were quantified (right) (*n =* 3). **b** Cell lysates were incubated with circRIMS2 or control probe, followed by WB. **c** The protein level of METTL3 was analyzed in N2a cells transfected with METTL3 (upper), and the results were quantified (lower) (*n =* 3). **d** The knockdown effect of shMETTL3 was tested in N2a cells 72 hours after transfection (*n =* 3). **e** The abundance of m6A-modified circRIMS2 was analyzed using MeRIP-PCR in N2a cells that were transfected with shMETTL3 after Aβ treatment (*n =* 3, one-way ANOVA with LSD post hoc). Scramble, Scramble + shRNA control; Aβ, Aβ + shRNA control; Aβ+shMETTL3, Aβ + shMETTL3. (Data are presented as mean ± S.E.M. and two-tailed t tests were used unless otherwise specified. **P* < 0.05, ***P* < 0.01, ****P* < 0.001, ^##^*P* < 0.01.)


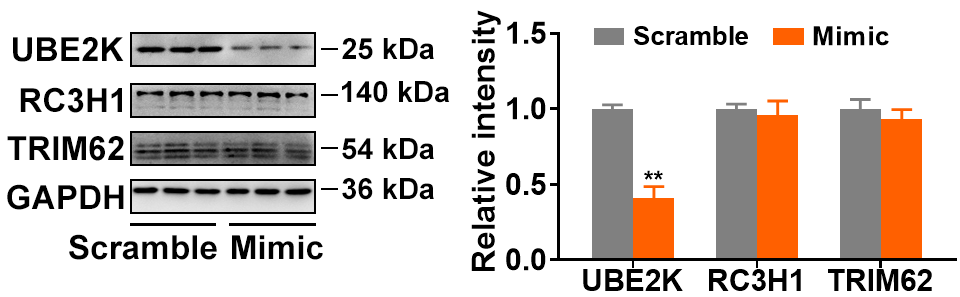


**Fig. S3 Downstream target validation of miR-3968.** The protein levels of UBE2K, RC3H1, and TRIM62 were measured in N2a cells transfected with scramble and miR-3968 Mimic (left). Quantitative analysis was performed (*n =* 3) (right).

(Data are presented as mean ± S.E.M. and two-tailed t tests were used unless otherwise specified. ***P* < 0.01.)


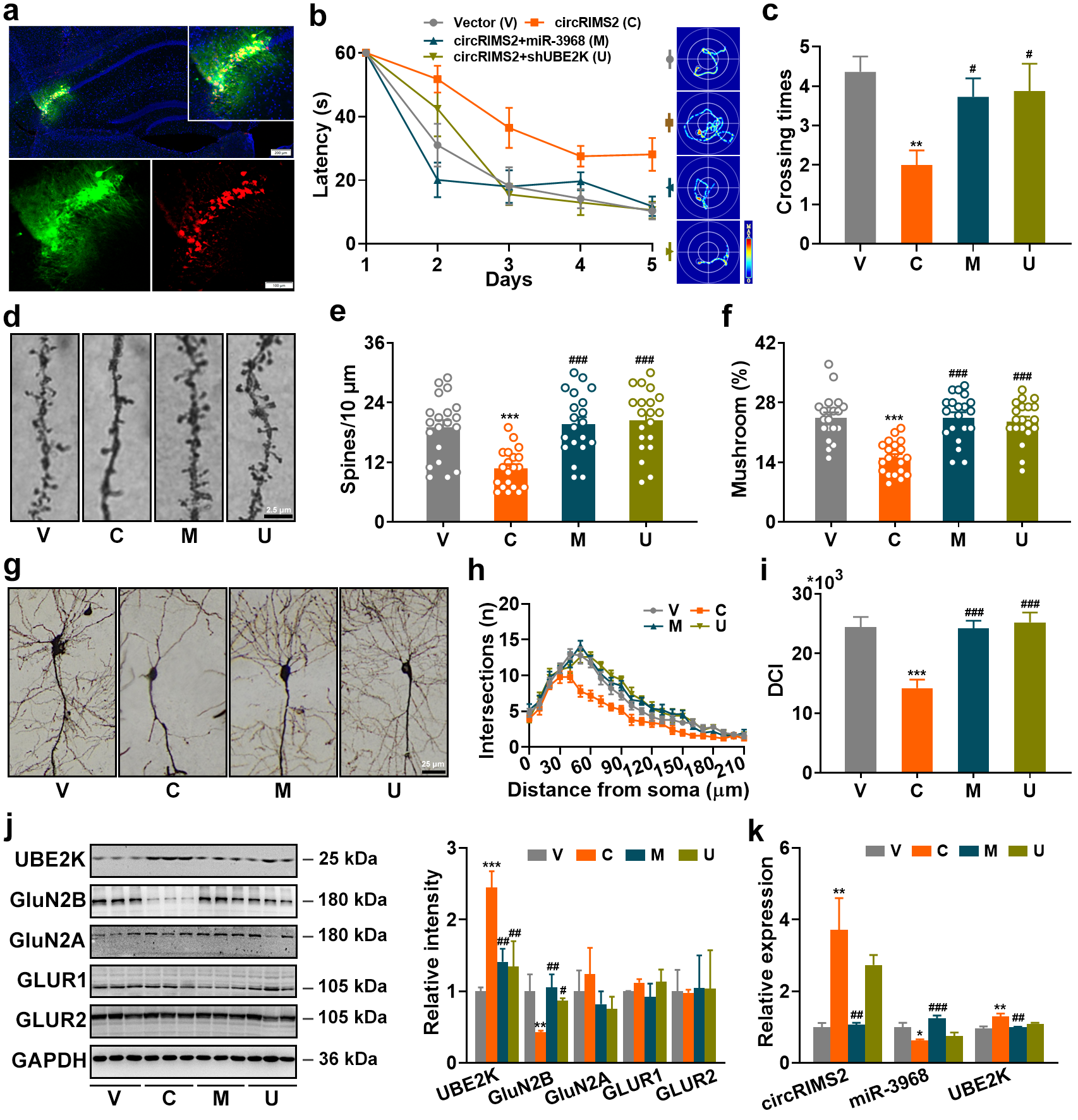


**Fig. S4 Overexpression of miR-3968 or silencing UBE2K rescues circRIMS2 induced memory impairment and synaptic disorders in vivo.** 4-month-old C57BL/6 mice were co-injected with circRIMS2 and miR-3968 or shUBE2K lentivirus into bilateral hippocampus. One month later, these mice underwent MWM, and then were sacrificed for WB, and Golgi-cox staining. The experimental groups were as follows: V: mice injected with vector lentivirus, C: mice injected with circRIMS2 lentivirus, M: mice co-injected with circRIMS2 and miR-3968 lentivirus, U: mice co-injected with circRIMS2 and shUBE2K lentivirus. **a** The lentivirus infected hippocampus slice was imaged for fluorescence. CircRIMS2 was labeled in green, while miR-3968 and shUBE2K lentiviruses were labeled in red. The CA3 areas were enlarged with a bar of 100 μm. **b, c** Performance of all mice was evaluated using MWM. Latency during the learning stage was recorded (**b**). The representative traces (**b**) and crossing times (**c**) on day 7 were examined. (*n =* 11, 12, 11,8 for V, C, M, U, one-way ANOVA with LSD post hoc). **d-f** Golgi staining was applied to demonstrate spine density and maturation. (**d**) Representative images of dendritic spines are shown Bar = 2.5 μm. Changes of spine density (per 10 μm, *n =* 20, one-way ANOVA with Tukey’s post hoc, **e**) and the percentage of mushroom-type spines (*n =* 20, one-way ANOVA with Tukey’s post hoc, **f**) are presented. **g-i** Dendritic morphology of neurons was analyzed using Golgi staining. (**g**) Representative images of dendritic trees are shown, with a scale bar of 25 μm. Sholl analysis (**h**) and DCI analysis (**i**) were used to examine dendritic complexity. **j** The protein levels of UBE2K, GluN2B, GluN2A, GLUR1 and GLUR2 were measured in hippocampus from four different groups using WB (left) and quantitative analysis was performed (right) (*n =* 3). **k** qRT-PCR analysis was performed to detect the expression of circRIMS2, miR-3968, and UBE2K in hippocampal homogenates from four different groups (*n =* 4). (Data are presented as mean ± S.E.M. and two-tailed t tests were used unless otherwise specified. **P* < 0.05, ***P* < 0.01, ****P*< 0.001, ^#^*P* < 0.05, ^##^*P* < 0.01, ^###^*P*< 0.001.)


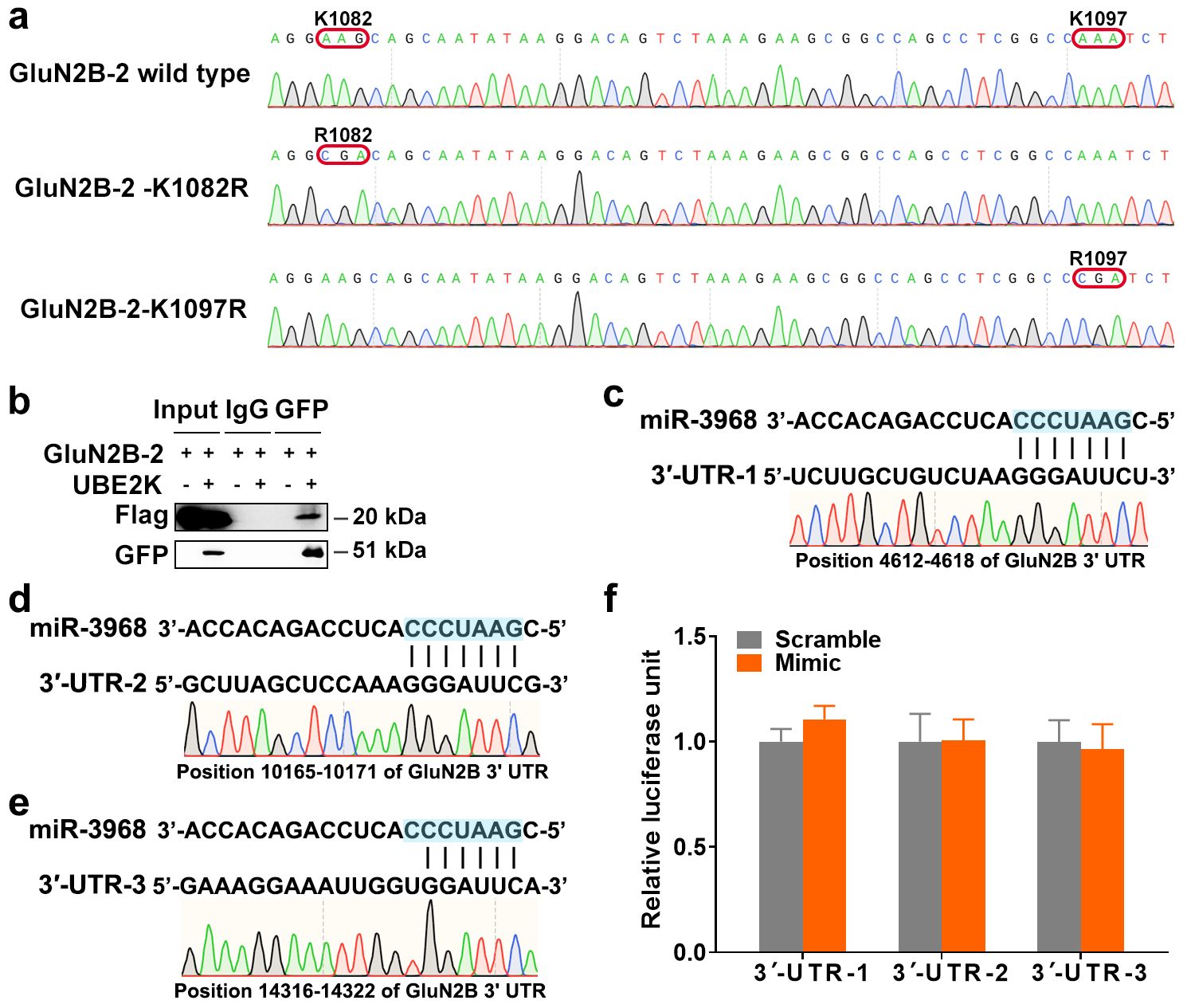


**Fig. S5 GluN2B-2 interacted with UBE2K. a** The K1082R and K1097R mutants of GluN2B-2 fragment were constructed and sequenced. **b** N2a cells were co-transfected with UBE2K-GFP and Flag-tagged GluN2B-2 fragment for 48 hours. Proteins were pulled down using IgG and anti-GFP antibodies and analyzed by WB using antibodies against GluN2B-2 (Flag) and UBE2K (GFP). **c-e** Three different predicted binding sites of miR-3968 within the 3′-UTR of GluN2B were constructed. **f** Dual-luciferase reporter assays revealed that miR-3968 mimic could not suppress the luciferase activity of WT 3′-UTR of GluN2B in HEK293 cells (*n =* 3). (Data are presented as mean ± S.E.M. and two-tailed t tests were used unless otherwise specified.)


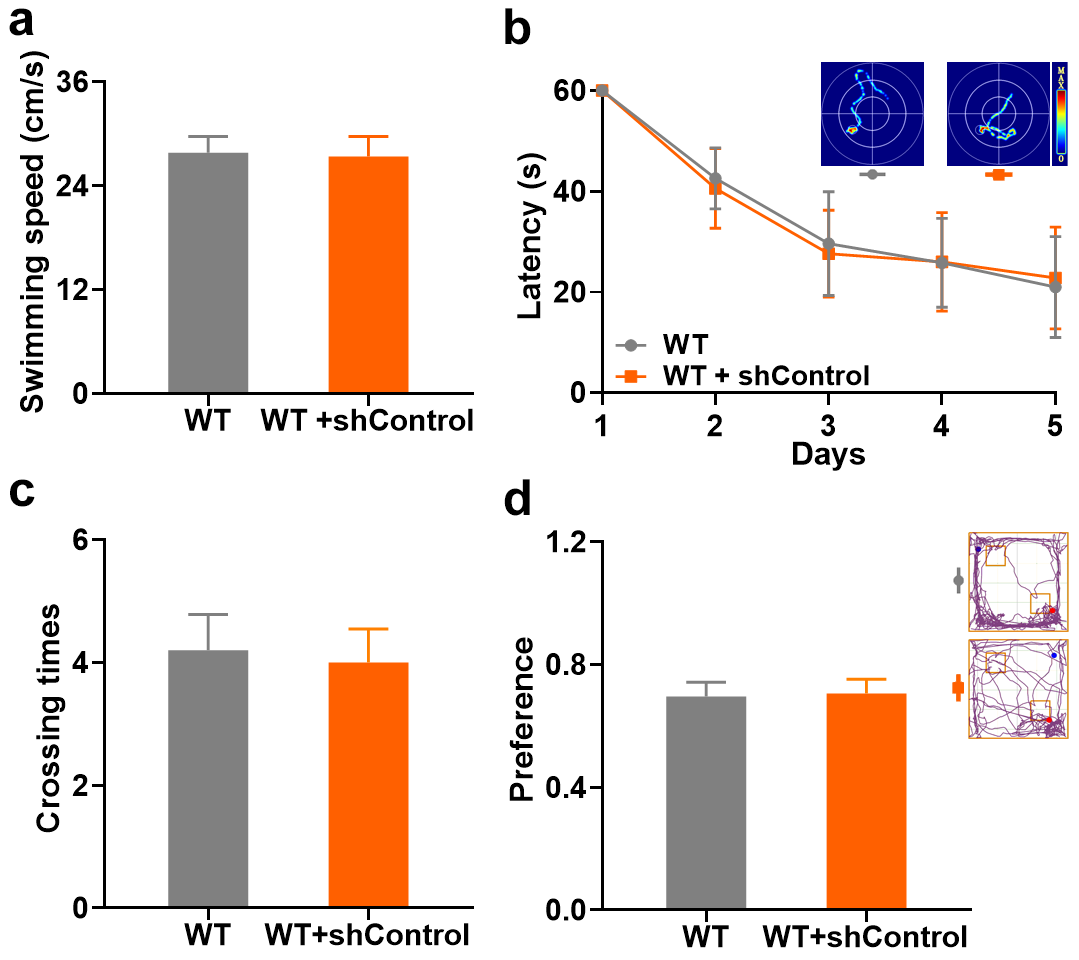


**Fig. S6 Injection of control lentivirus did not affect the learning and memory of WT mice.** WT mice were injected with or without lentivirus-shRNA Ctrl/-miRNA-Ctrl mix (1 μL for each). **a-c** Swimming speed, latency during the training period, and crossing times in test stage were analyzed. **d** Recognition memory was tested by NOR. No significant differences were found in swimming speed, latency, crossing times, and the preference index between WT mice and WT mice injected with control lentivirus (WT+shControl) (*n =* 5). (Data are presented as mean ± S.E.M. and two-tailed t tests were used unless otherwise specified.)


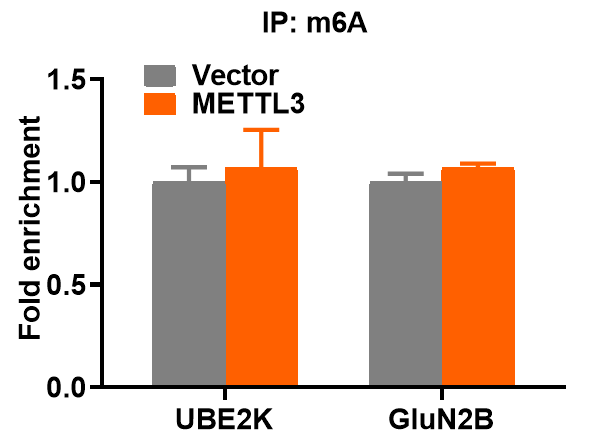


**Fig. S7 METTL3 did not affect the m6A modification of UBE2K and GluN2B.** N2a cells were transfected with METTL3 or Vector plasmid for 48 hours. MeRIP-PCR was applied to detect the m6A levels of UBE2K and GluN2B.


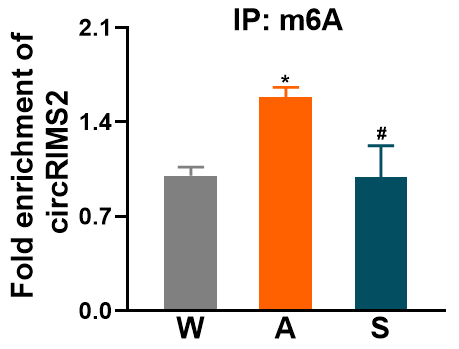


**Fig. S8 Silencing METTL3 reversed the m6A level of circRIMS2 in APP/PS1 mice.** The abundance of m6A-modified circRIMS2 was analyzed using MeRIP-PCR (*n =* 3, one-way ANOVA with LSD post hoc). The experimental groups were as follows: W - WT mice injected with shControl AAV, A - APP/PS1 mice injected with shControl AAV, S - APP/PS1 mice injected with shMETTL3 AAV. (Data are presented as mean ± S.E.M. and two-tailed t tests were used unless otherwise specified. **P* < 0.05, ^#^*P* < 0.05.)

**Supplementary Tables**

**Table S1 List of the primary and secondary antibodies**

| **Antibody** | **Host** | **Manufacturer** | **Cat #** |
| --- | --- | --- | --- |
| UBE2K | mouse | R&D | MAB6609 |
| UBE2K | rabbit | Proteintech | 11834-3-AP |
| GluN2B | rabbit | Proteintech | 21920-1-AP |
| GluN2B | mouse | Proteintech | 66565-1-Ig |
| GFP | rabbit | Proteintech | 50403-2-AP |
| Flag | mouse | Proteintech | 66008-3-Ig |
| GAPDH | mouse | Proteintech | 60004-1-Ig |
| actin-beta | mouse | Proteintech | 60008-1-Ig |
| METTL3 | rabbit | Proteintech | 15073-1-AP |
| ALKBH5 | rabbit | Bioswamp | PAB37455 |
| YTHDC1 | rabbit | Bioswamp | PAB31884 |
| IGF2BP1 | rabbit | Bioswamp | PAB31648 |
| RNF168 | rabbit | Bioswamp | PAB41048 |
| RNF138 | rabbit | Bioswamp | PAB36957 |
| RNF2 | rabbit | Zenbio | 382221 |
| GluN2A | rabbit | Abclonal | A19089 |
| GluA1 | rabbit | Abclonal | A11643 |
| GluA2 | rabbit | Abclonal | A11316 |
| SYN1 | rabbit | Abclonal | A17362 |
| SYP | rabbit | Abclonal | A6344 |
| PSD95 | rabbit | Abclonal | A7889 |
| Ubiquitin | mouse | Cell signaling | 3936 |
| Acetylated-lysine | mouse | Cell signaling | 9441 |
| CoraLite-488 Goat-anti-mouse IgG(H+L) | Goat | Proteintech | SA00013-1 |
| CoraLite-488 Goat-anti-rabbit IgG(H+L) | Goat | Proteintech | SA00013-2 |
| CoraLite-594 Goat-anti-mouse IgG(H+L) | Goat | Proteintech | SA00013-3 |
| CoraLite-594 Goat-anti-rabbit IgG(H+L) | Goat | Proteintech | SA00013-4 |
| Peroxidase-Conjugated Goat-anti-Mouse IgG(H+L) | Goat | Beyotime | A0216 |
| Peroxidase-Conjugated Goat-anti-Rabbit IgG(H+L) | Goat | Beyotime | A0208 |

**Table S2 The dysregulated circRNAs in the hippocampus of 4-month-old APP/PS1 mice.**

| **circRNA** | **log2FoldChange** | **P value** | **Type** | **Gene** |
| --- | --- | --- | --- | --- |
| 10:21345077-21354743:+ | Inf | 0.00559 | Up | HBS1L |
| 10:41220899-41240643:- | -Inf | 0.03025 | Down | FIG4 |
| 10:79860824-79862047:+ | 2.895058552 | 0.04388 | Up | PTBP1 |
| 10:88400652-88419560:+ | Inf | 0.04843 | Up | GNPTAB |
| 2:37469377-37475466:+ | 1.848139471 | 0.02893 | Up | RABGAP1 |
| 11:62117700-62132454:+ | Inf | 6.4E-06 | Up | SPECC1 |
| 11:80250208-80251126:+ | Inf | 0.04454 | Up | RHOT1 |
| 12:101883256-101890618:- | Inf | 0.01453 | Up | TRIP11 |
| 12:11271729-11290891:+ | 2.966757764 | 0.0441 | Up | SMC6 |
| 14:25502655-25581673:+ | 1.727898755 | 0.03591 | Up | ZMIZ1 |
| 9:66540062-66547955:- | 1.499311826 | 0.03657 | Up | USP3 |
| 12:52557489-52562427:+ | Inf | 0.02849 | Up | ARHGAP5 |
| 12:77331896-77332329:+ | 3.478415228 | 0.02708 | Up | FUT8 |
| 13:109439584-109442353:+ | Inf | 0.02011 | Up | PDE4D |
| 13:59460469-59482604:- | 2.362614397 | 0.03413 | Up | AGTPBP1 |
| 13:59460469-59544451:- | 3.771392743 | 0.00295 | Up | AGTPBP1 |
| 13:98784776-98789873:- | -Inf | 0.0458 | Down | FCHO2 |
| 14:101665761-101668576:+ | Inf | 0.01756 | Up | UCHL3 |
| 10:93235438-93236963:+ | 1.498855252 | 0.03442 | Up | CDK17 |
| 15:101198784-101204577:+ | Inf | 0.02987 | Up | ACVR1B |
| 15:20833297-20840805:+ | -Inf | 0.03189 | Down | N/A |
| 15:23382783-23410820:+ | Inf | 0.04454 | Up | CDH18 |
| 18:25339714-25344962:+ | 1.316451683 | 0.0322 | Up | AW554918 |
| 15:39436997-39535981:+ | 2.957390491 | 0.0352 | Up | RIMS2 |
| 15:81628293-81628737:+ | Inf | 0.04246 | Up | EP300 |
| 15:89246258-89253261:+ | -Inf | 0.04589 | Down | PPP6R2 |
| 15:90980795-90995633:- | -Inf | 0.02865 | Down | KIF21A |
| 16:10741614-10742931:+ | -Inf | 0.04467 | Down | CLEC16A |
| 16:89877038-89885125:- | 2.115195173 | 0.03524 | Up | TIAM1 |
| 17:37054131-37056979:+ | 2.529266656 | 0.02158 | Up | GABBR1 |
| 3:106531137-106539603:- | 1.308493392 | 0.01919 | Up | CEPT1 |
| 18:49877683-49894297:+ | Inf | 0.01283 | Up | DMXL1 |
| 18:53189592-53203119:+ | Inf | 0.02491 | Up | SNX2 |
| 18:5540606-5583796:- | 2.925222579 | 0.04237 | Up | GM10125 |
| 18:5748694-5773094:+ | -2.997308895 | 0.03211 | Down | ZEB1 |
| 18:69564037-69564684:+ | Inf | 0.04832 | Up | TCF4 |
| 18:77097249-77150098:+ | 2.612055365 | 0.02563 | Up | PIAS2 |
| 19:27849711-27982946:- | -Inf | 0.01406 | Down | RFX3 |
| 19:59305158-59327873:- | -Inf | 0.01946 | Down | PDZD8 |
| 1:10477025-10488949:- | Inf | 0.02229 | Up | CPA6 |
| 15:30634644-30683470:+ | -1.203021007 | 0.0474 | Down | CTNND2 |
| 1:167078537-167088619:+ | Inf | 0.04204 | Up | FAM78B |
| 1:170880778-170880932:+ | -Inf | 0.04378 | Down | DUSP12 |
| 1:21424049-21469564:- | -Inf | 0.04589 | Down | KCNQ5 |
| 1:25396436-25460811:- | 3.16903401 | 0.01836 | Up | ADGRB3 |
| 1:30829843-30831559:- | 2.647572898 | 0.0357 | Up | PHF3 |
| 1:39555478-39565332:- | -Inf | 0.02032 | Down | RNF149 |
| 1:66801049-66802168:- | 2.463330901 | 0.01027 | Up | KANSL1L |
| 2:104760494-104795294:- | Inf | 0.02874 | Up | QSER1 |
| 2:12242411-12265333:- | -Inf | 0.04166 | Down | ITGA8 |
| 2:146340379-146342749:- | Inf | 0.04083 | Up | RALGAPA2 |
| 2:156524881-156525288:+ | Inf | 0.03503 | Up | EPB41L1 |
| 9:96202131-96247088:+ | -1.271915702 | 0.04168 | Down | TFDP2 |
| 2:18159456-18171075:+ | 2.059490722 | 0.02412 | Up | MLLT10 |
| 2:25955260-25966948:- | -Inf | 0.04205 | Down | CAMSAP1 |
| 2:33052096-33064078:- | Inf | 0.01681 | Up | GARNL3 |
| 12:24878191-24934622:+ | -1.329357712 | 0.02357 | Down | MBOAT2 |
| 2:37532516-37543231:+ | Inf | 0.03253 | Up | RABGAP1 |
| 2:65670445-65690332:+ | Inf | 0.02033 | Up | SCN2A |
| 12:24854879-24882794:+ | -1.49880481 | 0.01955 | Down | MBOAT2 |
| 3:123627959-123672477:- | 1.648003327 | 0.04142 | Up | NDST3 |
| 3:127038747-127057091:- | 3.384297425 | 0.037 | Up | ANK2 |
| 3:136797726-136903228:+ | -Inf | 0.0398 | Down | PPP3CA |
| 3:32992906-33015076:- | Inf | 0.03699 | Up | PEX5L |
| 3:86532085-86542711:+ | -1.853274846 | 0.04007 | Down | LRBA |
| 4:107851308-107857320:+ | 1.8187821 | 0.02183 | Up | LRP8 |
| 4:108491348-108499398:+ | 3.21113836 | 0.02926 | Up | ZCCHC11 |
| 4:120982127-120985328:- | -Inf | 0.00644 | Down | SMAP2 |
| 4:128704998-128723713:+ | -Inf | 0.02048 | Down | PHC2 |
| 4:150500003-150570296:+ | -Inf | 0.04291 | Down | RERE |
| 4:155820329-155823693:+ | 3.174506807 | 0.0241 | Up | CCNL2 |
| 4:57204907-57225900:- | 2.970616366 | 0.03384 | Up | PTPN3 |
| 5:123733556-123740884:- | -Inf | 0.04418 | Down | RSRC2 |
| 5:140674337-140680945:- | -3.420499081 | 0.03507 | Down | IQCE |
| 5:18289524-18308825:- | 2.340298188 | 0.04286 | Up | GNAI1 |
| 5:19999442-20065671:+ | -2.550701476 | 0.03477 | Down | MAGI2 |
| 5:25281673-25293248:- | -Inf | 0.02782 | Down | KMT2C |
| 5:27382755-27469485:+ | Inf | 0.03238 | Up | DPP6 |
| 5:34419998-34440590:+ | Inf | 0.02504 | Up | FAM193A |
| 5:36922968-36931464:+ | Inf | 0.00476 | Up | PPP2R2C |
| 6:134506196-134542045:- | -Inf | 0.00943 | Down | LRP6 |
| 6:38286398-38287052:- | Inf | 0.01565 | Up | N/A |
| 6:54520700-54525711:- | Inf | 0.045 | Up | SCRN1 |
| 6:90689580-90694850:- | -1.549652948 | 0.03491 | Down | IQSEC1 |
| 7:132948044-132966694:+ | Inf | 0.00602 | Up | N/A |
| 7:132949585-132960734:+ | Inf | 0.01547 | Up | N/A |
| 7:14578736-14609339:- | -2.301307875 | 0.04879 | Down | NLRP5-PS |
| 7:29377496-29388151:- | Inf | 0.02033 | Up | SIPA1L3 |
| 7:59284026-59317973:- | Inf | 0.04498 | Up | SNHG14 |
| 7:59955645-59956800:- | -1.775867307 | 0.03421 | Down | SNHG14 |
| 7:62091114-62119043:- | Inf | 0.0414 | Up | N/A |
| 7:66908593-66915894:+ | -1.927076458 | 0.04157 | Down | ADAMTS17 |
| 7:96703926-96797505:+ | -2.253658042 | 0.0355 | Down | TENM4 |
| 8:125801461-125818810:- | Inf | 0.00099 | Up | PCNX2 |
| 8:17216607-17216823:- | -Inf | 0.03101 | Down | CSMD1 |
| 8:39046515-39097014:+ | -Inf | 0.04158 | Down | TUSC3 |
| 8:82068932-82071835:+ | Inf | 0.02033 | Up | INPP4B |
| 8:88156462-88158265:+ | Inf | 0.04183 | Up | HEATR3 |
| 8:95751741-95753150:- | 2.040215679 | 0.02647 | Up | CNOT1 |
| 9:100776756-100796855:+ | Inf | 0.04246 | Up | STAG1 |
| 9:104196023-104212904:- | Inf | 0.00921 | Up | DNAJC13 |
| 9:110072613-110079893:+ | -Inf | 0.00597 | Down | MAP4 |
| 9:32129570-32153124:+ | -2.244843299 | 0.00948 | Down | ARHGAP32 |
| 9:57019904-57027481:+ | -Inf | 0.0433 | Down | PTPN9 |
| 9:59801914-59874683:+ | 2.55674908 | 0.03622 | Up | MYO9A |
| 9:63281019-63303160:- | -2.246907625 | 0.02145 | Down | MAP2K5 |
| 9:64212584-64214609:- | Inf | 0.03102 | Up | MAP2K1 |
| 9:65373750-65377883:+ | Inf | 0.01037 | Up | UBAP1L |
| 1:165306178-165321814:+ | -2.030519565 | 0.03488 | Down | GPR161 |
| 9:72295279-72351570:+ | Inf | 0.02491 | Up | ZFP280D |
| 9:77239392-77243727:- | -Inf | 0.02809 | Down | MLIP |
| 9:9003620-9030872:- | Inf | 0.03228 | Up | ARHGAP42 |
| 9:95227188-95229200:+ | Inf | 0.03412 | Up | SLC9A9 |
| 2:17411086-17450310:- | -2.039409566 | 0.03666 | Down | NEBL |
| X:13093924-13103440:+ | -2.555277862 | 0.03461 | Down | USP9X |
| X:38402062-38442140:- | Inf | 0.0345 | Up | LAMP2 |

**Table S3 The dysregulated miRNAs in the hippocampus of 4-month-old APP/PS1 mice.**

| **miRNA** | **log2FoldChange** | **P value** | **Type** |
| --- | --- | --- | --- |
| mmu-miR-29b-1-5p | 0.868 | 0.02451 | Up |
| mmu-miR-448-3p | 0.54856 | 0.04055 | Up |
| mmu-miR-1258-3p | -2.0531 | 0.01286 | Down |
| mmu-miR-700-5p | 0.88847 | 0.02995 | Up |
| mmu-miR-10b-3p | 3.14377 | 0.02559 | Up |
| mmu-miR-615-3p | 4.98091 | 0.009 | Up |
| mmu-miR-194-5p | 0.86493 | 0.04872 | Up |
| mmu-miR-196b-5p | 2.45626 | 0.04355 | Up |
| mmu-miR-10a-3p | 3.72342 | 0.01298 | Up |
| mmu-miR-200a-5p | -0.8436 | 0.03608 | Down |
| mmu-let-7j | 0.59885 | 0.04769 | Up |
| mmu-miR-2137 | -4.0697 | 0.02526 | Down |
| mmu-miR-429-3p | -0.8946 | 0.01078 | Down |
| mmu-miR-192-5p | 0.77195 | 0.02913 | Up |
| mmu-miR-133b-3p | 1.53504 | 0.01818 | Up |
| mmu-miR-201-5p | 1.29275 | 0.04512 | Up |
| mmu-miR-3968 | -0.6167 | 0.02605 | Down |
| mmu-miR-200a-3p | -0.6769 | 0.02622 | Down |
| mmu-miR-320-3p | 0.82015 | 0.02011 | Up |
| mmu-miR-10b-5p | 3.28447 | 0.00225 | Up |
| mmu-miR-10a-5p | 2.1539 | 0.01468 | Up |
| mmu-miR-195a-3p | 1.15739 | 0.02266 | Up |
| mmu-miR-196a-5p | 3.59739 | 0.02337 | Up |
| mmu-miR-1912-3p | 0.73701 | 0.01156 | Up |
| mmu-miR-3473a | -2.0563 | 0.04266 | Down |
| mmu-miR-3963 | -1.1328 | 0.04515 | Down |
| mmu-miR-547-3p | 1.00652 | 0.01398 | Up |
| mmu-miR-497a-3p | 3.57121 | 0.01524 | Up |
| mmu-miR-3535 | 0.7755 | 0.03509 | Up |

**Table S4 The predicted circRNA/miRNA ceRNA pairs by miRanda.**

| **circRNA** | **miRNA** | **circRNA change** | **circRNA p value** | **miRNA change** | **miRNA p value** | **correlation** | **correlation  p value** |
| --- | --- | --- | --- | --- | --- | --- | --- |
| circRNA-1 | miR-200a-3p | 2.967 | 0.044 | -0.677 | 0.026 | -0.928 | 0.0077 |
| circRNA-1 | miR-200a-3p | 2.557 | 0.036 | -0.677 | 0.026 | -0.899 | 0.0149 |
| circRNA-3 | miR-3473a | 3.478 | 0.027 | -2.056 | 0.043 | -0.812 | 0.0499 |
| circRNA-4 | miR-3968 | 2.957 | 0.035 | -0.617 | 0.026 | -0.812 | 0.0498 |
| circRNA-5 | miR-3968 | 3.771 | 0.003 | -0.617 | 0.026 | -0.986 | 0.0003 |
| circRNA-6 | miR-29b-1-5p | -1.550 | 0.035 | 0.868 | 0.025 | -0.886 | 0.0333 |
| circRNA-7 | miR-29b-1-5p | -1.853 | 0.040 | 0.868 | 0.025 | -0.943 | 0.0167 |
| circRNA-8 | miR-196a-5p | -2.551 | 0.035 | 3.597 | 0.023 | -0.851 | 0.0317 |
| circRNA-9 | miR-10a-3p | -1.499 | 0.020 | 3.723 | 0.013 | -0.812 | 0.0499 |
| circRNA-8 | miR-196b-5p | -2.551 | 0.035 | 2.456 | 0.044 | -0.836 | 0.0382 |
| circRNA-10 | miR-497-3p | -2.254 | 0.035 | 3.571 | 0.015 | -0.899 | 0.0149 |
| circRNA-8 | miR-497a-3p | -2.551 | 0.035 | 3.571 | 0.015 | -0.882 | 0.0199 |
| circRNA-9 | miR-497a-3p | -1.499 | 0.020 | 3.571 | 0.015 | -0.899 | 0.0149 |
| circRNA-11 | miR-497a-3p | -2.039 | 0.037 | 3.571 | 0.015 | -0.986 | 0.0003 |

Correlation was calculated using Pearson analysis. circRNA-1: 12:11271729-11290891:+; circRNA-2: 9:59801914-59874683:+; circRNA-3: 12:77331896-77332329:+; circRNA-4 (circRIMS2): 15:39436997-39535981:+; circRNA-5: 13:59460469-59544451:-; circRNA-6: 6:90689580-90694850:-; circRNA-7: 3:86532085-86542711:+; circRNA-8: 5:19999442-20065671:+; circRNA-9: 12:24854879-24882794:+; circRNA-10: 7:96703926-96797505:+; circRNA-11: 2:17411086-17450310:-.

**Table S5. The predicted targets of miR-3968.**

| **TargetScan** | **miRDB** | **microT-CDS** | **Intersected** |
| --- | --- | --- | --- |
| MSR1 | SFTPA1 | GCLC | ESCO2 |
| RP11-386G21.2 | DERL2 | 4930455H04RIK | SEMA4A |
| CYP4F22 | GRIA4 | TMEM241 | TRIM62 |
| FAM188A | RAB31 | CHRD | JMY |
| C12orf50 | STAM2 | HES7 | VAPB |
| KLHL13 | LYN | JRKL | SPOP |
| LAMTOR5 | SMC3 | ZMAT3 | HEYL |
| SLMO1 | PLA2G4C | TFAP2E | ABCG1 |
| RP11-215A19.2 | ZBTB24 | SP100 | MIER1 |
| RP11-724O16.1 | SRPX | TFAP2C | PIP5K1A |
| UPB1 | IL1RAP | SRSF3 | RICTOR |
| ZNF420 | KMT5B | GM6483 | RC3H1 |
| CTB-96E2.2 | FBXW8 | UQCC1 | NOS1 |
| VPS45 | PGM2L1 | TMEM67 | TMEM43 |
| KLHL41 | NUFIP2 | SOWAHA | RASAL2 |
| C1orf141 | CCDC167 | ZDHHC3 | SELE |
| CYB561D1 | GSX2 | COL27A1 | FOXP2 |
| ZNF226 | PTPN4 | RBP4 | DAAM1 |
| IL18R1 | LRP4 | COL16A1 | UBE2K |
| ZNF660 | ADGRD1 | RPS15A | TXNDC17 |
| CA11 | BAK1 | RCC2 | EIF5 |
| CDKN2B | TSC22D4 | ACSM3 |  |
| LRRC3DN | HRASLS | TRMT12 |  |
| TSPAN7 | NCF4 | RORA |  |
| C3orf70 | CLNS1A | WRB |  |
| TBXAS1 | TMEM260 | ACER2 |  |
| C2orf66 | GM45927 | SLC6A2 |  |
| PAX8 | CAPS2 | MMS19 |  |
| DNAJB12 | CNIH1 | HSD17B13 |  |
| PTAFR | TRIM45 | XPNPEP1 |  |
| CEND1 | TTC23 | KAZN |  |
| CYSLTR2 | FRAT2 | KCNC2 |  |
| FAM60A | PLAG1 | SLC16A12 |  |
| EOMES | NAALADL2 | FBXL19 |  |
| PPP1R3D | RDH19 | 2610044O15RIK8 | |
| HECA | GAS2L1 | NTNG1 |  |
| SYT10 | NAT8F4 | TRP63 |  |
| OSBP | PEG12 | B630019K06RIK |  |
| HTR2A | 2510009E07RIK | C77370 |  |
| C1QTNF7 | RDH9 | GPATCH2 |  |
| SPRN | PARP1 | HSF5 |  |
| CLDN10 | PRELID3A | TRIAP1 |  |
| MIER2 | NXPE2 | PCTP |  |
| CCDC50 | SGPP2 | SLC30A4 |  |
| ZNF324 | RAB43 | 1700012B07RIK |  |
| FLRT1 | QTRT2 | ITGA7 |  |
| DFFB | PHB | SLC4A3 |  |
| SDR16C5 | LRBA | PEX11B |  |
| SLC26A10 | MMD | GOSR1 |  |
| ZNF213 | RDH1 | LOXL1 |  |
| IL2RB | NPHP4 | CYP2C66 |  |
| SOCS3 | MLST8 | HIPK1 |  |
| IL2RA | CMTM2A | ALOX8 |  |
| GTF2H1 | SAMD4 | HDDC3 |  |
| C6orf89 | TTC33 | CYP2C65 |  |
| MEGF11 | CASP4 | CDK8 |  |
| EMP2 | DOC2B | GM10097 |  |
| MCIDAS | KCNN1 | NLGN2 |  |
| RAB27B | CHADL | KLF7 |  |
| CLSTN2 | ZMAT4 | ELF2 |  |
| MBD1 | TYW3 | TRIM16 |  |
| CDK2 | KCNA6 | SUSD3 |  |
| AK4 | TRPM3 | METTL3 |  |
| NID1 | TMEM178 | SRP54B |  |
| DLX6 | SPHKAP | PCDHB14 |  |
| HRK | SHC2 |  |  |
| FAM47E | TPRN |  |  |
| TMEM229B | DUS4L |  |  |
| FLG2 | KRT86 |  |  |
| MYOZ2 | CGAS |  |  |
| CALHM1 | GABRG1 |  |  |
| MAPK4 | TSPOAP1 |  |  |
| RAD51L3-RFFL | POFUT2 |  |  |
| GPR37L1 | SLC29A3 |  |  |
| SF1 | GPR82 |  |  |
| POLR3G | AKR1C14 |  |  |
| PALM2 | PPFIA4 |  |  |
| MAML2 | TRPC2 |  |  |
| ST8SIA4 | INO80D |  |  |
| GXYLT1 | WNT16 |  |  |
| KCTD15 | XNTRPC |  |  |
| RFFL | MBNL3 |  |  |
| PFKFB2 | E2F1 |  |  |
| SRL | ZFP445 |  |  |
| CMKLR1 | COQ3 |  |  |
| NDNF | SAA4 |  |  |
| SLC5A10 | XPR1 |  |  |
| PIF1 | CLEC7A |  |  |
| GDF7 | TMEM45A2 |  |  |
| RLIM | PTPRB |  |  |
| ANKS4B | ARPC2 |  |  |
| RAB11FIP2 | GPX5 |  |  |
| RANBP10 | TIMD2 |  |  |
| MGAM | SLITRK6 |  |  |
| SCYL3 | PKDREJ |  |  |
| QTRTD1 | ELL |  |  |
| ANGPT1 | MBNL2 |  |  |
| CELF5 | MRPL15 |  |  |
| C1orf233 | RBM3 |  |  |
| NAA25 |  |  |  |
| AP1G1 |  |  |  |
| SPATA17 |  |  |  |
| ENO2 |  |  |  |
| ARID4A |  |  |  |
| SEC16A |  |  |  |
| HCN1 |  |  |  |
| SIM1 |  |  |  |
| RAI1 |  |  |  |
| SSR3 |  |  |  |
| TIA1 |  |  |  |
| TXNDC5 |  |  |  |
| TMEM108 |  |  |  |
| ADCY9 |  |  |  |
| EIF4EBP2 |  |  |  |
| SAMD4B |  |  |  |
| GJA3 |  |  |  |
| MLEC |  |  |  |
| FAM124A |  |  |  |
| TIMM10B |  |  |  |
| ANKRD34C |  |  |  |
| AGPAT5 |  |  |  |
| NOS1AP |  |  |  |
| SCN2B |  |  |  |
| AKAP2 |  |  |  |
| OGFOD2 |  |  |  |
| PALM2-AKAP2 |  |  |  |
| ARHGAP26 |  |  |  |
| SLC2A14 |  |  |  |
| CHD6 |  |  |  |
| TLL2 |  |  |  |
| C20orf112 |  |  |  |
| MYNN |  |  |  |
| CREB5 |  |  |  |
| ZFP3 |  |  |  |
| SUV420H1 |  |  |  |
| GCLM |  |  |  |
| AR |  |  |  |
| METTL2B |  |  |  |
| IQSEC3 |  |  |  |
| TAF9B |  |  |  |
| DERL3 |  |  |  |
| XKR7 |  |  |  |
| POU4F1 |  |  |  |
| BBS9 |  |  |  |
| SLC27A4 |  |  |  |
| DIP2C |  |  |  |
| GABBR2 |  |  |  |
| ZNF324B |  |  |  |
| ATF7IP |  |  |  |
| CMTM6 |  |  |  |
| ZCCHC14 |  |  |  |
| CBX6 |  |  |  |
| SP1 |  |  |  |
| EDC3 |  |  |  |
| FREM1 |  |  |  |
| CD38 |  |  |  |
| ARHGAP18 |  |  |  |
| ELK4 |  |  |  |
| SHFM1 |  |  |  |
| DNMT3A |  |  |  |
| PAWR |  |  |  |
| IST1 |  |  |  |
| TMEM192 |  |  |  |
| SLC8A1 |  |  |  |
| DGKH |  |  |  |
| FOXA1 |  |  |  |
| UBN2 |  |  |  |
| ARL5B |  |  |  |
| CASC10 |  |  |  |
| KCNJ6 |  |  |  |
| POU6F1 |  |  |  |
| TNKS |  |  |  |
| ZNF280D |  |  |  |
| NTN1 |  |  |  |
| LIN7A |  |  |  |
| SP9 |  |  |  |
| MPDZ |  |  |  |
| NUDT7 |  |  |  |
| SLC38A1 |  |  |  |
| NABP1 |  |  |  |
| RIMS2 |  |  |  |
| AGTPBP1 |  |  |  |
| LRP3 |  |  |  |
| PPP3CC |  |  |  |
| C9orf64 |  |  |  |
| APPBP2 |  |  |  |
| GREM1 |  |  |  |
| ARL1 |  |  |  |
| RSBN1L |  |  |  |
| GPR98 |  |  |  |
| PHKB |  |  |  |
| IP6K1 |  |  |  |
| GTDC1 |  |  |  |
| PKIA |  |  |  |
| SMNDC1 |  |  |  |
| LYRM4 |  |  |  |
| EIF2B2 |  |  |  |
| MTHFR |  |  |  |
| USP14 |  |  |  |
| SLC25A4 |  |  |  |
| PIGP |  |  |  |
| AKR1C4 |  |  |  |
| PIN1 |  |  |  |
| DHX30 |  |  |  |
| C14orf119 |  |  |  |
| CHST1 |  |  |  |
| JUND |  |  |  |
| RPS6KL1 |  |  |  |
| ZBTB20 |  |  |  |
| METTL2A |  |  |  |
| PCK1 |  |  |  |
| CNTNAP5 |  |  |  |
| CCNB1 |  |  |  |
| RYK |  |  |  |
| MYC |  |  |  |
| SNX17 |  |  |  |
| INSIG2 |  |  |  |
| CDC5L |  |  |  |
| TLR4 |  |  |  |
| COL1A1 |  |  |  |
| SYTL4 |  |  |  |
| LIMK2 |  |  |  |
| SHC3 |  |  |  |
